# Flexible direct regeneration of heterogeneous cathode materials of spent lithium-ion batteries at industrial scale

**DOI:** 10.1093/nsr/nwag017

**Published:** 2026-01-10

**Authors:** Junxiong Wang, Guanjun Ji, Haocheng Ji, Song Liu, Junfeng Li, Yanfei Zhu, Nengzhan Zheng, Zheng Liang, Guangmin Zhou, Hui-Ming Cheng

**Affiliations:** Tsinghua Shenzhen International Graduate School, Tsinghua University, Shenzhen 518055, China; Frontiers Science Center for Transformative Molecules, School of Chemistry and Chemical Engineering, Shanghai Jiao Tong University, Shanghai 200240, China; Frontiers Science Center for Transformative Molecules, School of Chemistry and Chemical Engineering, Shanghai Jiao Tong University, Shanghai 200240, China; Tsinghua Shenzhen International Graduate School, Tsinghua University, Shenzhen 518055, China; Frontiers Science Center for Transformative Molecules, School of Chemistry and Chemical Engineering, Shanghai Jiao Tong University, Shanghai 200240, China; Tsinghua Shenzhen International Graduate School, Tsinghua University, Shenzhen 518055, China; Tsinghua Shenzhen International Graduate School, Tsinghua University, Shenzhen 518055, China; Tsinghua Shenzhen International Graduate School, Tsinghua University, Shenzhen 518055, China; Frontiers Science Center for Transformative Molecules, School of Chemistry and Chemical Engineering, Shanghai Jiao Tong University, Shanghai 200240, China; Tsinghua Shenzhen International Graduate School, Tsinghua University, Shenzhen 518055, China; Institute of Technology for Carbon Neutrality, Shenzhen Institute of Advanced Technology, Chinese Academy of Sciences, Shenzhen 518055, China; Shenyang National Laboratory for Materials Science, Institute of Metal Research, Chinese Academy of Sciences, Shenyang 110016, China

**Keywords:** direct recycling, cathode materials, ambient condition, large-scale application

## Abstract

Direct recycling has emerged as a promising alternative to existing recycling methods due to its simplicity and cost-effectiveness. However, its scalable application remains a subject of debate, primarily due to the complexity of mixed degraded cathode materials in practice. The reason is that degraded materials with different compositions are extremely difficult to be repaired to produce cathode materials with uniform composition and performance. Herein, we have successfully realized direct regeneration of mixed heterogeneous degraded LiNi_0.5_Co_0.2_Mn_0.3_O_2_ from different sources on an industrial scale. First, uniform contact lithiation is achieved through the van der Waals force between Li-1-methyl-2-pyrrolidinone and LiNi_0.5_Co_0.2_Mn_0.3_O_2_ molecules, leaving them in a uniform lithium-rich state. A self-saturating synthetic lithiation process occurs during subsequent heating, ensuring that each particle from various sources is repaired as needed. This method has been demonstrated to treat 50 kg of cathode materials per batch, and the regenerated products show uniform and excellent performance, achieving a retention rate of 90.7% after 1500 cycles in Ah-level pouch cells. This performance is the best result reported to date and sets a new benchmark for regenerated LiNi_x_Co_y_Mn_1-x-y_O_2_ cathode materials, which have reached the standard for direct commercial use.

## Teaser

This study achieved large-scale direct recycling of actual end-of-life battery materials through a unique reaction mechanism and method design, with the resulting regenerated materials meeting commercial application standards.

## INTRODUCTION

Direct recycling of degraded cathode materials of lithium-ion batteries has evolved significantly in recent years, and a variety of methods have been validated in the laboratory with a single type of cathode material [1,2]. However, a critical challenge remains: during repair, it is often necessary to determine the amount of supplementary lithium by first analyzing the components of a degraded cathode material. Though this approach is reasonable in experimental studies, it becomes impractical in practical applications because actual degraded cathode materials are mixtures obtained from disassembled batteries with varying states of health (SOH) [3,4]. Even if the compositional supplementation could be performed according to the average value of the measured composition, it fails to ensure homogeneous single particle-level restoration due to intrinsic SOH variation. These fundamental limitations have raised substantial doubts about the scalability of direct recycling technologies, with no comprehensive industrial-scale demonstrations reported to date [5].

The most common existing direct recycling method is solid-state sintering, in which lithium salts are mechanically mixed with degraded cathode materials followed by high-temperature treatment [6]. However, due to the varying sizes and morphologies of the solid particles, achieving a uniform distribution of lithium salts at the particle scale is challenging. As a result, the effectiveness of this method in treating heterogeneous cathode materials has been less than satisfactory [7]. Compared to solid-state reactions, solid–liquid reactions offer more uniform contact between reactants. However, these reactions often require heating and pressurization [8] (hydrothermal method) or electrification [9] (electrochemistry method) conditions to provide the necessary driving force for lithium ion diffusion. This results in complex equipment, and makes the process less suitable for large-scale applications.

In order to treat heterogeneous cathode materials with different SOH, it is necessary to ensure complete lithiation of each individual particle [10]. This process should be carried out by solid–liquid reactions and requires a small amount of additional energy input [11,12]. In this case, the lack of a driving force makes it difficult for lithium ions in solution to re-intercalate directly into the degraded cathode materials by diffusion. This led us to consider an alternative: why not make the lithium exist in a solution in a molecular form, and utilize the affinity of certain functional groups to specific atoms in the cathode materials to facilitate spontaneous contact? [7] The whole process does not involve redox reactions and can be carried out relatively quickly, resulting in the formation of a lithium-rich domain on the surface of each particle [13]. Subsequently, through heating, lithium in these surface lithium-rich domains re-intercalates into the degraded cathode materials through a synthetic chemical reaction, rather than by diffusion, as is typical with lithium in a solution [14]. Since a high-temperature lithiation process involves a synthetic reaction, the lithiation capacity during this stage becomes self-regulated by the intrinsic lithium deficiency of each particle [15]. As long as the surface of each particle is guaranteed to be in a lithium-rich state, on-demand repair of particles with different compositions can ideally be realized.

In this study, we present a liquid-phase regeneration method based on LiOH–*N*-methyl-2-pyrrolidinone (LiOH–NMP) molecules, comprising two key steps, i.e. contact lithiation and synthetic lithiation. We systematically demonstrate the scheme of LiOH–NMP molecules and its interactions with the molecules of cathode materials during the contact lithiation process, as well as the self-saturation reaction mechanism during the synthetic lithiation process. The effectiveness of this method has been validated in a series of experiments, ranging from several grams to 50 kg. The resulting massive regenerated cathode materials exhibit uniform and excellent performance, with a retention rate of 93.1% after 1000 cycles in 1.8 Ah pouch cells, the best performance of the regenerated LiNi_0.5_Co_0.2_Mn_0.3_O_2_ (NCM523) cathode materials reported so far, and it has already reached the requirements for practical commercial applications. This study marks the first systematic demonstration of the scalable direct recycling of heterogeneous NCM523 cathode materials, setting a milestone for the future development of direct recycling technologies.

## RESULTS

### Formation of LiOH–NMP and its interaction with cathode materials

**Figure 1.**
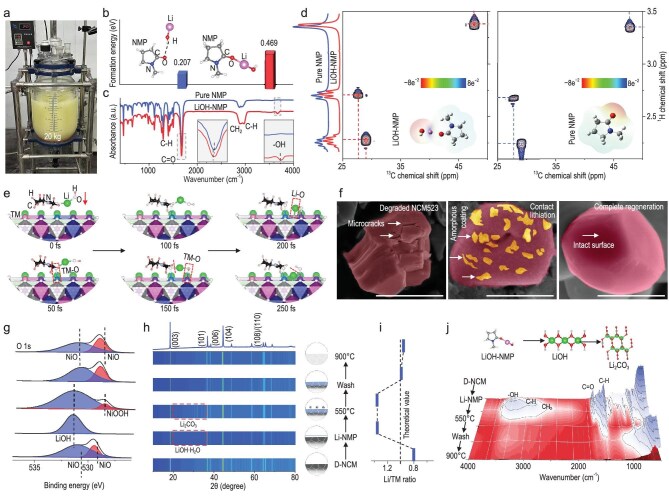
Formation of LiOH–NMP and its interaction with cathode materials. (a) Photo of a LiOH–NMP solution obtained by batch production. (b) Formation energies of different binding forms of LiOH and NMP molecules. (c) Comparison of IR spectra of NMP molecules before and after binding to LiOH. (d) Comparison of 2D NMR spectra of NMP molecules before and after binding to LiOH. (e) AIMD results of the interaction between LiOH–NMP and NCM523 cathode molecules. (f–j) Changes in the physical and chemical properties of the degraded NCM523 during the repair process: (f) morphology (scale bar = 1 μm); (g) O 1s XPS spectra; (h) XRD patterns; (i) inductively coupled plasma (ICP) results; (j) IR spectra.

The first step in this regeneration process is the preparation of an LiOH–NMP solution, which was obtained by dissolving LiOH in NMP solution at room temperature and pressure (Fig. 1a). The binding form of LiOH and NMP in the solution is crucial for the subsequent mechanism analysis and therefore needs to be clarified first. Based on previous literature, we hypothesized two possible configurations of the LiOH–NMP complex [16,17], and calculated their corresponding formation energies. We found that the formation energy is higher (0.469 eV) when Li in LiOH binds to O on the C=O group in NMP, so the expected molecular structure is shown in Fig. 1b. Infrared (IR) spectroscopy confirmed this bonding, as an additional –OH peak appeared after the combination of LiOH and NMP, compared to pure NMP [18] (Fig. 1c). The combination of LiOH and NMP also affects the electron distribution of the NMP molecules, especially the introduced –OH, which affects the electron cloud near the H atom, which is manifested in the observed chemical shift of the hydrogen signal in the nuclear magnetic resonance (NMR) spectrum [16,19] (Fig. 1d). This result proves that the combination is not a simple dissolution, but rather the formation of specific molecular compounds in solution, which is crucial for the subsequent contact lithiation process.

After determining the molecular configuration, we used *ab* initio molecular dynamics (AIMD) to simulate possible interactions between the NCM523 and LiOH–NMP. The results showed that after bonding with Li, the oxygen in the C=O groups of NMP easily interacts with the transition metal atoms in NCM523, forming a bond that grafts the LiOH molecules onto the NCM523 surface. This interaction occurs spontaneously without the need for external force (such as heating). The LiOH–NMP formation is subsequently disrupted, leaving LiOH molecules on the surface of the cathode particles (Fig. 1e). This is confirmed by scanning electron microscopy (SEM) results, where the degraded cathode materials, upon contact with an LiOH–NMP solution at ambient conditions, forms uniformly distributed scales on the surface, which disappear after subsequent heating (Fig. 1f, Figs S1 and S2). X-ray photoelectron spectroscopy (XPS), X-ray diffraction (XRD) patterns and XRD refinement further confirm that these scale-like amorphous substances are LiOH (Fig. 1g and h, Fig. S3). In contrast, when solid LiOH is directly mixed with cathode materials, the contact is very uneven due to the lack of interaction forces between them (Fig. S4).

Once the contact lithiation is complete, the material needs to be heated to complete the regeneration process. Thermogravimetric analysis (TGA) revealed that the weight loss during the repair process showed different patterns below and above 550°C, so we used this as a demarcation point to investigate the physical phase and compositional changes during the heating process. It was found that the Li/transition metal (TM) ratio of the degraded cathode materials increased from approximately 0.8 to about 1.0 after heating at 550°C (Fig. 1i). This is very interesting because we also observed the transition of LiOH to Li_2_CO_3_ at lower temperatures (Fig. 1h and j, Fig. S5 and Tables S1 and S2). It is known that LiOH is first converted to Li_2_CO_3_ and then decomposed into Li_2_O at higher temperatures (approximately 700°C, Fig. S6) [20], where it re-intercalates into the degraded cathode material and achieves compositional replenishment [21]. The results here demonstrate that the compositional replenishment is completed below 550°C, representing a novel discovery. The lithiation process at lower temperatures plays a crucial role in the direct repair of mixed cathode materials, which will be discussed in detail later.

However, the supplementation of the composition does not mean the full recovery of electrochemical performance. We analyzed the material after the treatment at 550°C and found that the electrochemical performance has not been restored (Fig. S7). It can only be completely repaired after the subsequent high temperature treatment at 900°C, and the understanding of this problem must start from the structural analysis of the material.

### Formation of localized lithium-rich regions during direct regeneration

**Figure 2.**
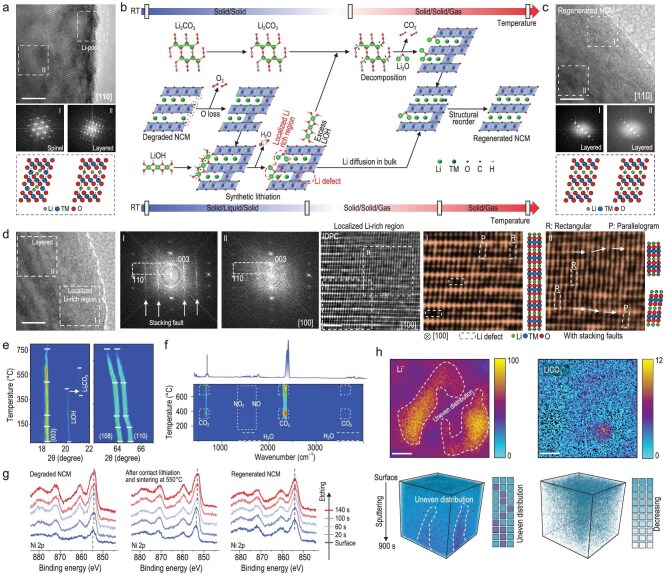
Formation of localized lithium-rich regions during direct regeneration. (a) Analysis of TEM results of degraded NCM523 materials. (b) Schematic of the difference between the regeneration path proposed in this paper and the common solid-state sintering path. RT: room temperature. (c) Analysis of TEM results of regenerated NCM523 materials. (d) Evidence from TEM observations of localized lithium-rich regions within the material during repair. (e) *In situ* XRD patterns during the heating process. (f) Thermogravimetric (TG)-IR results during the heating process. (g) In-depth XPS analysis of NCM samples at different states. (h) TOF-SIMS analysis of samples in localized lithium-rich states.

The gradual loss of the active lithium component in a degraded material after prolonged cycling leads to a phase transition (Fig. 2a, Fig. S8), as reported in numerous studies [22,23]. However, as we mentioned earlier, after heating at 550°C, the overall composition of the material is restored to its initial state, but its performance is not. Therefore, it can be reasonably presumed that, although lithium re-intercalates into the cathode material at lower temperatures through synthetic lithiation, it does not accurately occupy the lithium sites. This suggests that, in this intermediate state, lithium is unevenly distributed within the cathode material, likely forming lithium-rich regions (Fig. 2b). After subsequent heating, the lithium migrates and rearranges within the bulk, eventually forming a regular layered structure (Fig. 2c). In order to confirm the existence of these intermediate lithium-rich regions, the structure of the samples sintered at 550°C was analyzed in detail. With the help of selected area electron diffraction (SAED), and integrated differential phase contrast (iDPC) scanning transmission electron microscopy (STEM) techniques, we clearly observed lithium-rich structural features attributed to the stacking fault of lithium [24,25]. This SAED pattern could be explained by the superposition of three SAED patterns corresponding to the Li_2_MnO_3_-type (Li-rich phase) structure (*C2/m*) in different orientations. Furthermore, the presence of microscale lithium-rich regions, which caused lattice distortions, was observed along the [100] direction (Fig. 2d, Fig. S9) [26]. The occurrence of two different atomic arrangements (rectangular and parallelogram) in localized regions is a typical characteristic of the lithium-rich Li_2_MnO_3_ phase. This confirms that during the repair process, Li^+^ re-intercalating into the material at lower temperatures does not precisely occupy the Li sites, but rather forms a localized uneven distribution of lithium concentration, resulting in Li-poor and Li-rich regions.

This phenomenon is further supported by in-depth XPS analysis (Figs S10–S12), which reveals a significant difference in the nickel (Ni) valence between the surface and the bulk of the degraded cathode materials. After lithiation and heat treatment at 550°C, even though the composition has been recovered, a difference in the Ni valence persists between the near-surface regions and the bulk. After the high-temperature treatment, the Ni valence throughout the whole particle is the same (Fig. 2g). The Raman spectroscopy also confirms that lithiation occurred below 550°C (Fig. S13). The change in Ni valence is directly related to the concentration distribution of Li, as shown by time-of-flight secondary ion mass spectrometry (TOF-SIMS), which directly indicates a local concentration difference within the bulk [27,28] (Fig. 2h). The uneven distribution of lithium is the main reason why the composition of the material is recovered but the performance is not fully recovered. To achieve complete regeneration, atomic rearrangement of lithium and transition metals within the bulk is required, which can only be achieved through further diffusion at high temperatures. This underscores the importance of the subsequent high-temperature treatment (900°C).

In addition, through the *in situ* XRD observation of the synthetic lithiation process, it can be preliminarily inferred that the lithiation process started at approximately 220°C, because the (108) and (110) peaks firstly showed an obvious merging tendency, which is caused by an intrinsic phase transition of degraded materials after heating [29], but this merging tendency stopped above 220°C (Fig. 2e). It can be inferred that the lithiation process began at this point and hindered the phase transition of the degraded cathode material itself due to heating. The conversion of LiOH to Li_2_CO_3_ can also be clearly observed between 400°C and 600°C [21], supporting our earlier hypothesis that the synthetic lithiation process is carried out directly by the degraded NCM523 with LiOH at lower temperatures, rather than through the intermediate conversion to Li_2_CO_3_ and subsequently to Li_2_O. Correspondingly, more CO_2_ is detected after heating to 400°C and 600°C, relating to the decomposition of the binder [30] attached to the degraded cathode materials (Fig. 2f) and the thermal decomposition of Li_2_CO_3_ converted by the excess LiOH on the cathode surface (Fig. 2h).

### Validation of this direct regeneration method for heterogeneous cathode materials

**Figure 3.**
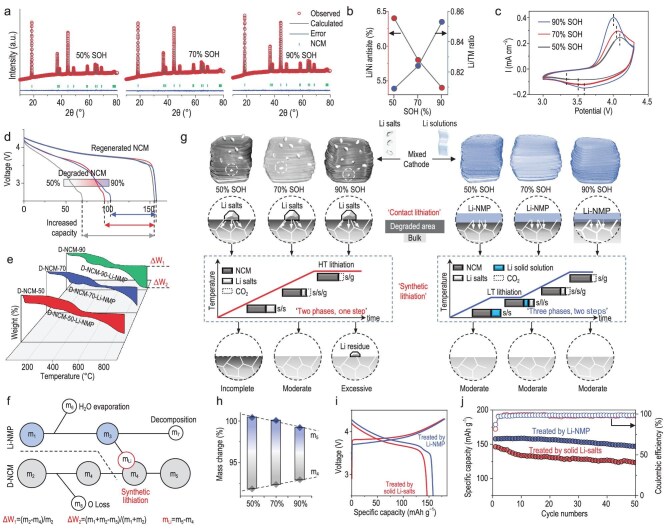
Validation of the direct regeneration method for heterogeneous cathode materials. (a) XRD refinement patterns of degraded NCM523 with different SOH. (b) Changes in the degree of Li/Ni antisites and the Li/TM molar ratio with the SOH. (c) Cyclic voltammetry (CV) curves of degraded NCM523 with different SOH. (d) Residual capacity of degraded NCM523 with different SOH and their capacity after repair. (e) Comparison of TG curves of degraded NCM523 with different SOH before and after mixing with LiOH–NMP. (f) Calculation procedure to derive the amount of lithium absorbed by the synthetic lithiation process by means of TG curves. (g) Comparison of the effect of direct regeneration using direct solid-phase mixing with the proposed method for mixed cathode materials. (h) Changes in lithium content absorbed during synthetic lithiation of degraded cathode materials with different SOH. (i) Charging and discharging curves, and (j) cycling performances of regenerated mixed cathode materials treated by LiOH and LiOH–NMP.

As discussed earlier, we have elaborated the repair mechanism of the degraded cathode materials through contact and synthetic lithiation. Since the mass involved in the reaction during the lithiation process is determined by the reactants themselves, we propose that this process exhibits a self-saturation behavior, i.e. the amount of lithium that the cathode material can absorb is determined by the amount of its own lithium loss, which is crucial for the treatment of mixed cathode materials. To confirm this, we assembled pouch cells with commercial cathode materials, cycled them for an extended period, and then disassembled them to obtain degraded cathode materials with different SOH. Structural analysis revealed that the degree of failure correlates with an increased number of Li–Ni antisite defects, leading to poorer electrochemical performance (Fig. 3a–c, Tables S3–S6). We used the same reaction conditions (with the same lithium dosage) to repair three kinds of cathode materials with different residual capacities (74.0, 98.8 and 106.0 mAh g^−1^), and their specific capacities were basically restored to a similar level (approximately 155 mAh g^−1^), which provides preliminary confirmation of the self-saturated repair process (Fig. 3d, Fig. S14).

To confirm this self-saturation behavior, it is necessary to analyze the mass change during synthetic lithiation. The degraded cathode material, due to its structural defects, is highly susceptible to oxygen loss when heated [31], resulting in a reduction of its own mass (Fig. 3e), and the weight loss of a material during heating increases with the degree of failure. When the contact lithiation is completed, the surface of the material is in a lithium-rich state, and part of the lithium is re-intercalated into the material by the synthetic lithiation, while the remaining part is volatilized by heat. Therefore, the difference of mass change between the initial state and the state after contact lithiation can be used to determine the amount of lithium involved in synthetic lithiation (Fig. 3f). Calculations show that the amount of lithium absorbed by the synthetic lithiation process increases with the degree of failure, which directly confirms our inference (Fig. 3h, Fig. S15).

We homogeneously mixed three kinds of degraded cathode materials and repaired them, comparing the results with direct solid-state sintering using lithium salts. Our method shows a significantly better effect than direct solid-state sintering (Fig. 3i and j). This improvement can be attributed to the uniformity of the contact in our approach. Solid-phase contact cannot guarantee that each particle is in a uniform lithium-rich state, leading to variability in the repair of individual particles, which ultimately affects the overall performance. Another reason is that during solid-state sintering, lithium salts need to be decomposed first at higher temperatures before being re-intercalated into the material; the volatilization process of lithium salts and the synthetic lithiation occur at the same time. This may cause lithium to evaporate completely from the surface of the cathode material before it can be completely absorbed, resulting in incomplete repair (Fig. 3g). As mentioned earlier, synthetic lithiation of the material started at about 220°C, and the lithium had already re-intercalated into the crystal lattice at 550°C. This ensures that no lithium volatilizes during the subsequent high-temperature process, preventing uneven recovery. This is the importance of the lithiation process being carried out at lower temperatures for material repair. Based on these results, we have elucidated the repair mechanism of this method in detail and confirmed its feasibility for direct repair of mixed cathode materials.

### Direct regeneration of actual heterogeneous degraded cathode materials at industrial scale

**Figure 4.**
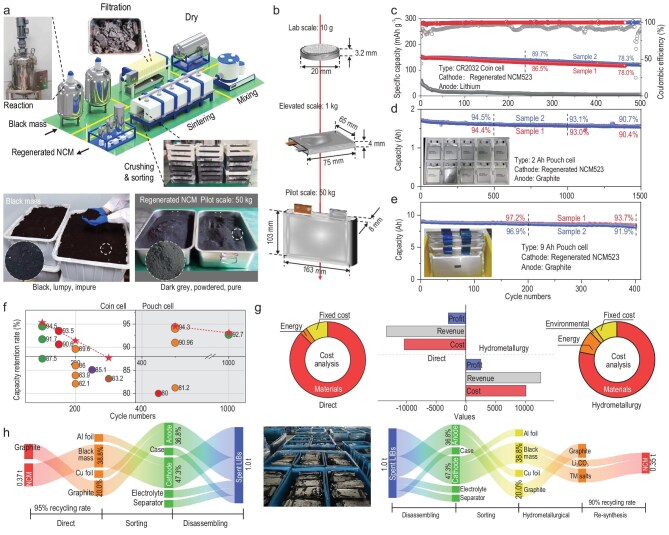
Direct regeneration of actual heterogeneous degraded cathode materials at industrial scale. (a) Production-line design for direct regeneration of actual mixed cathode materials, and photos of equipment, raw materials and products. (b) Cell parameters used to verify the performance of regenerated products at different production scales. (c–e) Performance of cells prepared from regenerated products at different production scales: (c) 10 g scale; (d) 1 kg scale; and (e) 50 kg scale. (f) Comparison of capacity retention rates of previous reports with this work; (g and h) Comparison of procedures and economic analysis of the hydrometallurgical method with this work.

In order to verify the repair effect of the present method on real-world heterogeneous cathode materials, we conducted a series of scaled-up experiments in a pilot plant (Fig. S16) with several tonnes of degraded cathode materials of complex origin, the so-called black mass, purchased directly from the market (Fig. 4a). First, a 10 g-level validation was carried out in the laboratory, and the regenerated cathode materials were assembled into coin cells with an initial capacity of approximately 155 mAh g^−1^ at 0.5 C (Fig. S17), and the retention rate was close to 80% after 500 cycles, demonstrating homogeneous battery performance (Fig. 4b and c, Fig. S18). Subsequent validations were conducted at larger scales: 1 and 50 kg, using a prototype production line designed by our team (Fig. 4a). After assembling the kg-scale regenerated products into pouch cells, the performance consistency between different cells was excellent, and the cycle performance was high, with a retention rate of 93.1% after 1000 cycles (Fig. 4d, Fig. S19). In the subsequent 50 kg-scale experiment, the regenerated products were prepared into larger-capacity pouch cells, achieving a retention rate of 93.7% for 400 cycles (Fig. 4e). The cycling performance of both coin cells and pouch cells made from the regenerated product is the best reported for NCM cathode materials [32–49] (Fig. 4f, Table S7). The cycling performance of a 1.8 Ah pouch cell has almost reached the standard for direct commercial use. This is the first time in direct recycling research that a systematic scale-up application study has been reported and a scale-up product validation has been made.

Based on the data obtained from our actual pilot-scale experiments, we performed a concise economic analysis (Fig. 4g and h). Since the raw materials treatment in the direct recycling method is essentially the same as the hydrometallurgical process, we primarily compare the two. The direct recycling process has simpler steps than the hydrometallurgical process (Fig. S20), thus the overall recovery rate is slightly higher (Fig. 4h). Although the hydrometallurgical process has similar cost ($10118.3) compared with direct recovery ($9900.7), the recovered product needs to be resynthesized to cathode materials. Therefore, on the whole, the direct recycling method generates much more revenue ($13364.1) from processing 1 tonne of black mass than the hydrometallurgical process ($12621.65) (Fig. 4g). As the price of lithium salts is gradually reduced, the material cost of direct recycling will be gradually reduced, while for the hydrometallurgical process, lithium salts are its product, indicating that the value of its product will be gradually reduced, therefore, the economic advantage of direct recycling over the hydrometallurgical process will be more obvious in the future.

## DISCUSSION

After several years of development, direct recycling needs to gradually move from the laboratory to practical applications. The complexity of the source of degraded cathode materials is one of the major challenges that direct recycling must overcome. In this paper, we propose a unique strategy of constructing a lithium-rich environment on the surface through contact lithiation and subsequently completing the regeneration by synthetic lithiation under heating. The homogeneous lithium-rich environment on the surface ensures that each particle in the mixed cathode materials is in full contact with the lithium source, and the synthetic lithiation process guarantees that the composition of each particle can be restored to its initial state due to the self-saturation nature of the synthetic reaction. Based on the complex black mass obtained from the dismantling of actual spent lithium-ion batteries, we successfully regenerated 50 kg of cathode materials in one batch with excellent uniform performance, reaching a level suitable for direct commercial application. Pilot-scale tests for general cathode manufacturers typically involve batches of 50–100 kg, and this is the first reported case of direct recycling of NCM cathode materials at actual production scale. This achievement marks a significant milestone for the practical application of direct recycling.

Although the performance of our regenerated products has been validated, their actual commercial application still requires further development. The first is the control of raw material impurities. Although a small amount of impurities such as aluminum has proved to be beneficial to the cycling performance, in commercial applications, cathode material manufacturers still want to have as few impurities as possible, which requires further optimization of both the dismantling process and the regeneration process. In addition, the practical application scenarios and value assessment of regenerated materials also need to be promoted with the cooperation of policymakers, producers and users. Direct recycling has an inherent advantage in the technical principle, and the problems faced by its practical application are gradually being solved, so it is believed that direct recycling will achieve real-scale commercial application in the near future.

## METHODS

### The regeneration processes

Degraded cathode materials are first washed with NaOH solution (1 M) to remove aluminum impurities, followed by the repair process, which consists of two main steps, namely contact lithiation and synthetic lithiation. LiOH–NMP solution was prepared in the contact lithiation process: LiOH was dissolved in an analytically pure NMP solution, stirring at room temperature under room pressure until the color of the solution changed to pale yellow, and the powder was basically dissolved; the solid–liquid ratio of the reaction was about 1:50. The degraded cathode materials were then added to the solution while stirring at room temperature. The specific solid–liquid ratio does not need to be precisely controlled as long as the amount of lithium in the solution exceeds the amount of lithium required to repair the degraded cathode material according to its components.

After 4 h of reaction, the solid was separated from the solution and subsequently dried in an oven at 60°C. The dried powder was then mixed with 3 wt% of Li_2_CO_3_ to complete the synthetic lithiation process by heating, first from room temperature to 550°C for 2 h, and then increasing to 900°C for 2 h, at a rate of 5°C min^−1^ in air. A small amount of Li_2_CO_3_ was mixed to supplement the loss of lithium due to high-temperature sintering. The sintered material was ground and then washed alternately with water and ethanol to remove residual alkali from the surface and then dried and heat at 500°C for 2 h to obtain the final regenerated cathode materials.

### Theoretical calculation

The DFT calculations were performed using the Vienna *Ab initio* Simulation Package (VASP) [50–52]. The generalized gradient approximation (GGA) with the Perdew–Burke–Ernzerhof (PBE) [53] exchange-correlation functional was employed. Projector augmented-wave (PAW) pseudopotentials [54] were utilized to model core electron interactions, with a plane-wave energy cut-off of 500 eV. Supercell geometries were optimized with convergence criteria set to 1 × 10^−5^ eV for energy and 0.02 eV/Å for force. Gaussian smearing, with a width of 0.05 eV, was applied in both geometry optimizations and total energy calculations. The calculations were carried out using 5 × 4 supercells (60 formula units, R${\bar{3}}$m space group) of an α-NaFeO_2_-type structure. From this bulk structure, the NCM523 (100) crystallographic plane was cleaved to model the NCM523 surface. For enhanced accuracy in describing electronic interactions, GGA + U calculations were performed with empirically determined U values, as suggested by previous studies on similar materials [55,56]. Specifically, U values of 5.96, 5.00 and 5.10 eV were assigned to Ni, cobalt (Co) and manganese (Mn), respectively. Finally, the interaction of the NCM523 surface with the LiOH–NMP was studied through AIMD simulations, performed within the NVT ensemble at 300 K using a 1 fs time step on the optimized surface model.

Due to length constraints, some experimental details have been placed in the Supplementary data (including Figs S21–S25 and Tables S8–S13).

## Supplementary Material

nwag017_Supplemental_File

## References

[1] Ma X, Meng Z, Bellonia MV et al. The evolution of lithium-ion battery recycling. Nat Rev Clean Technol 2025; 1: 75–94. 10.1038/s44359-024-00010-4

[2] Harper G, Sommerville R, Kendrick E et al. Recycling lithium-ion batteries from electric vehicles. Nature 2019; 575: 75–86. 10.1038/s41586-019-1682-5 31695206

[3] Roy JJ, Phuong DM, Verma V et al. Direct recycling of Li-ion batteries from cell to pack level: challenges and prospects on technology, scalability, sustainability, and economics. Carbon Energy 2024; 6: e492. 10.1002/cey2.492

[4] Wang J, Ma J, Zhuang Z et al. Toward direct regeneration of spent lithium-ion batteries: a next-generation recycling method. Chem Rev 2024; 124: 2839–87. 10.1021/acs.chemrev.3c00884 38427022

[5] Hayagan N, Gaalich I, Loubet P et al. Challenges and perspectives for direct recycling of electrode scraps and end-of-life lithium-ion batteries. Batter Supercaps 2024; 7: e202400120. 10.1002/batt.202400120

[6] Ji H, Wang J, Ma J et al. Fundamentals, status and challenges of direct recycling technologies for lithium ion batteries. Chem Soc Rev 2023; 52: 8194–244. 10.1039/D3CS00254C 37886791

[7] Wang J, Ji H, Li J et al. Direct recycling of spent cathode material at ambient conditions via spontaneous lithiation. Nat Sustain 2024; 7: 1283–93. 10.1038/s41893-024-01412-9

[8] Yu X, Yu S, Yang Z et al. Achieving low-temperature hydrothermal relithiation by redox mediation for direct recycling of spent lithium-ion battery cathodes. Energy Storage Mater 2022; 51: 54–62. 10.1016/j.ensm.2022.06.017

[9] Arnold S, Ruthes JG, Kim C et al. Electrochemical recycling of lithium-ion batteries: advancements and future directions. EcoMat 2024; 6: e12494. 10.1002/eom2.12494

[10] Lin J, Li W, Chen Z. Scaling direct recycling of lithium-ion batteries toward industrialization: challenges and opportunities. ACS Energy Lett 2025; 10: 947–57. 10.1021/acsenergylett.4c03176

[11] Gupta V, Yu X, Gao H et al. Scalable direct recycling of cathode black mass from spent lithium-ion batteries. Adv Energy Mater 2023; 13: 2203093. 10.1002/aenm.202203093

[12] Li J, Shi R, Wang J et al. Interfacial metal-solvent chelation for direct regeneration of LiFePO_4_ cathode black mass. Adv Mater 2025; 37: 2414235. 10.1002/adma.202414235 39629549

[13] Shi R, Zheng N, Ji H et al. Homogeneous repair of highly degraded Ni-rich cathode material with spent lithium anode. Adv Mater 2024; 36: 2311553. 10.1002/adma.202311553 38124361

[14] Choi G, Chang U, Lee J et al. Unraveling and regulating superstructure domain dispersion in lithium-rich layered oxide cathodes for high stability and reversibility. Energy Environ Sci 2024; 17: 4634–45. 10.1039/D4EE00487F

[15] Xiao X, Wang L, Li J et al. Rational synthesis of high-performance Ni-rich layered oxide cathode enabled via probing solid-state lithiation evolution. Nano Energy 2023; 113: 108528. 10.1016/j.nanoen.2023.108528

[16] Karak S, Singh H, Biswas A et al. Lithiophilic dibenzamide linkages to impart lithium storage capacity in porous polybenzamides. J Am Chem Soc 2024; 146: 20183–92. 10.1021/jacs.4c05192 39002137

[17] Lu F, Zhang C, Lu B et al. Cellobiose as a model compound for cellulose to study the interactions in cellulose/lithium chloride/*N*-methyl-2-pyrrolidone systems. Cellulose 2017; 24: 1621–9. 10.1007/s10570-017-1213-1

[18] Sun D, Zhang J, Ren H et al. Influence of OH^−^ and SO_4_^2−^ anions on morphologies of the nanosized nickel hydroxide. J Phys Chem C 2010; 114: 12110–6. 10.1021/jp1033849

[19] Zhang C, Liu R, Xiang J et al. Dissolution mechanism of cellulose in *N,N*-dimethylacetamide/lithium chloride: revisiting through molecular interactions. J Phys Chem B 2014; 118: 9507–14. 10.1021/jp506013c 25026263

[20] Wang J, Ma J, Jia K et al. Efficient extraction of lithium from anode for direct regeneration of cathode materials of spent Li-ion batteries. ACS Energy Lett 2022; 7: 2816–24. 10.1021/acsenergylett.2c01539

[21] Chen W, Li J, Ji H et al. Efficient and scalable direct regeneration of spent layered cathode materials via advanced oxidation. Adv Mater 2025; 37: 2416818. 10.1002/adma.202416818 39806841

[22] Jia K, Wang J, Zhuang Z et al. Topotactic transformation of surface structure enabling direct regeneration of spent lithium-ion battery cathodes. J Am Chem Soc 2023; 145: 7288–300. 10.1021/jacs.2c13151 36876987

[23] Jia K, He Y, Piao Z et al. Low-frequency phonon dispersion relation enabling stable cathode from spent lithium-ion batteries. Adv Mater 2025; 37: 2413753. 10.1002/adma.202413753 39707693

[24] Ito A, Li D, Sato Y et al. Cyclic deterioration and its improvement for Li-rich layered cathode material Li[Ni_0.17_Li_0.2_Co_0.07_Mn_0.56_]O_2_. J Power Sources 2010; 195: 567–73. 10.1016/j.jpowsour.2009.07.052

[25] Serrano-Sevillano J, Reynaud M, Saracibar A et al. Enhanced electrochemical performance of Li-rich cathode materials through microstructural control. Phys Chem Chem Phys 2018; 20: 23112–22. 10.1039/C8CP04181D 30168545

[26] Lei C, Bareno J, Wen J et al. Local structure and composition studies of Li_1.2_Ni_0.2_Mn_0.6_O_2_ by analytical electron microscopy. J Power Sources 2008; 178: 422–33. 10.1016/j.jpowsour.2007.11.077

[27] Xie C, Zhao C, Jeong H et al. Regulating Li nucleation and growth heterogeneities via near-surface lithium-ion irrigation for stable anode-less lithium metal batteries. Small 2024; 20: 2306868. 10.1002/smll.202306868 37946620

[28] Jin B, Dolocan A, Liu C et al. Regulating anode-electrolyte interphasial reactions by zwitterionic binder chemistry in lithium-ion batteries with high-nickel layered oxide cathodes and silicon-graphite anodes. Angew Chem Int Ed 2024; 63: e202408021. 10.1002/anie.202408021 39019796

[29] Jung S-K, Kim H, Song S et al. Unveiling the role of transition-metal ions in the thermal degradation of layered Ni–Co–Mn cathodes for lithium rechargeable batteries. Adv Funct Mater 2022; 32: 2108790. 10.1002/adfm.202108790

[30] Li P, Xu H, Luo S et al. Green and non-destructive separation of cathode materials from aluminum foil in spent lithium-ion batteries. Sep Purif Technol 2024; 338: 126625. 10.1016/j.seppur.2024.126625

[31] Sharifi-Asl S, Lu J, Amine K et al. Oxygen release degradation in Li-ion battery cathode materials: mechanisms and mitigating approaches. Adv Energy Mater 2019; 9: 1900551. 10.1002/aenm.201900551

[32] Guo Y, Liao X, Huang P et al. High reversibility of layered oxide cathode enabled by direct re-generation. Energy Storage Mater 2021; 43: 348–57. 10.1016/j.ensm.2021.09.016

[33] Guo Y, Guo C, Huang P et al. Rejuvenating LiNi_0.5_Co_0.2_Mn_0.3_O_2_ cathode directly from battery scraps. eScience 2023; 3: 100091. 10.1016/j.esci.2023.100091

[34] Qian G, Li Z, Wang Y et al. Value-creating upcycling of retired electric vehicle battery cathodes. Cell Rep Phys Sci 2022; 3: 100741. 10.1016/j.xcrp.2022.100741

[35] Zhang Y, Yao N, Tang X et al. Upcycling of high-rate Ni-rich cathodes through intrinsic structural features. Adv Energy Mater 2024; 14: 2402918. 10.1002/aenm.202402918

[36] Mancini M, Hoffmann M, Martin J et al. A proof-of-concept of direct recycling of anode and cathode active materials: from spent batteries to performance in new Li-ion cells. J Power Sources 2024; 595: 233997. 10.1016/j.jpowsour.2023.233997

[37] Yang T, Luo D, Zhang X et al. Sustainable regeneration of spent cathodes for lithium-ion and post-lithium-ion batteries. Nat Sustain 2024; 7: 776–85. 10.1038/s41893-024-01351-5

[38] Ko S, Choi J, Hong J et al. Thermodynamically controlled chemical regeneration of spent battery cathodes using recyclable electron donors under ambient conditions. Energy Environ Sci 2024; 17: 4064–77. 10.1039/D3EE04528E

[39] Guo Y, Li Y, Qiu K et al. Removal of residual contaminants by minute-level washing facilitates the direct regeneration of spent cathodes from retired EV Li-ion batteries. Energy Environ Sci 2025; 18: 264–74. 10.1039/D4EE03021D

[40] Xiao Z, Yang Y, Li Y et al. Strong oxidizing molten salts for strengthening structural restoration enabling direct regeneration of spent layered cathode. Small 2024; 20: 2309685. 10.1002/smll.202309685 38238155

[41] Fan M, Meng X, Guo H et al. Reviving fatigue surface for solid-state upcycling of highly degraded polycrystalline LiNi_1-x-y_Co_x_Mn_y_O_2_ cathodes. Adv Mater 2024; 36: 2405238. 10.1002/adma.202405238 38923661

[42] Fan M, Chang X, Guo Y et al. Increased residual lithium compounds guided design for green recycling of spent lithium-ion cathodes. Energy Environ Sci 2021; 14: 1461–8. 10.1039/D0EE03914D

[43] Wang T, Luo H, Bai Y et al. Direct recycling of spent nickel-rich cathodes in reciprocal ternary molten salts. J Power Sources 2024; 593: 233798. 10.1016/j.jpowsour.2023.233798

[44] Xing C, Da H, Yang P et al. Aluminum impurity from current collectors reactivates degraded NCM cathode materials toward superior electrochemical performance. ACS Nano 2023; 17: 3194–203. 10.1021/acsnano.3c00270 36724114

[45] Liu Y, Jiang W, Ling M et al. Revealing lithium configuration in aged layered oxides for effective regeneration. ACS Appl Mater Interfaces 2023; 15: 9465–74. 10.1021/acsami.2c21841 36753671

[46] Tong H, Lv H, Li Y et al. Bifunctional treatment of spent ternary cathode materials with improved electrochemical performance. ACS Appl Energy Mater 2024; 7: 2816–24. 10.1021/acsaem.3c03263

[47] Qin Z, Zhang Y, Luo W et al. A universal molten salt method for direct upcycling of spent Ni-rich cathode towards single-crystalline Li-rich cathode. Angew Chem Int Ed 2023; 62: e202218672. 10.1002/anie.202218672 37083044

[48] Fan X, Tan C, Li Y et al. A green, efficient, closed-loop direct regeneration technology for reconstructing of the LiNi_0.5_Co_0.2_Mn_0.3_O_2_ cathode material from spent lithium-ion batteries. J Hazard Mater 2021; 410: 124610. 10.1016/j.jhazmat.2020.124610 33243647

[49] Huang C, Xia X, Chi Z et al. Preparation of single-crystal ternary cathode materials via recycling spent cathodes for high performance lithium-ion batteries. Nanoscale 2022; 14: 9724–35. 10.1039/D2NR00993E 35762909

[50] Kresse G, Hafner J. *Ab initio* molecular-dynamics simulation of the liquid-metal–amorphous-semiconductor transition in germanium. Phys Rev B 1994; 49: 14251–69. 10.1103/PhysRevB.49.14251 10010505

[51] Kresse G, Joubert D. From ultrasoft pseudopotentials to the projector augmented-wave method. Phys Rev B 1999; 59: 1758–75. 10.1103/PhysRevB.59.1758

[52] Kresse G, Furthmüller J. Efficiency of ab-initio total energy calculations for metals and semiconductors using a plane-wave basis set. Comput Mater Sci 1996; 6: 15–50. 10.1016/0927-0256(96)00008-0 9984901

[53] Perdew J, Burke K, Ernzerhof M. Generalized gradient approximation made simple. Phys Rev Lett 1996; 77: 3865–8. 10.1103/PhysRevLett.77.3865 10062328

[54] Blöchl P . Projector augmented-wave method. Phys Rev B 1994; 50: 17953. 10.1103/physrevb.50.17953 9976227

[55] Dixit M, Markovsky B, Aurbach D et al. Unraveling the effects of Al doping on the electrochemical properties of LiNi_0.5_Co_0.2_Mn_0.3_O_2_ using first principles. J Electrochem Soc 2017; 164: A6359–65. 10.1149/2.0561701jes

[56] Schipper F, Dixit M, Kovacheva D et al. Stabilizing nickel-rich layered cathode materials by a high-charge cation doping strategy: zirconium-doped LiNi_0.6_Co_0.2_Mn_0.2_O_2_. J Mater Chem A 2016; 4: 16073–84. 10.1039/C6TA06740A

