# Supplementary material for: Flexible direct regeneration of heterogeneous cathode materials of spent lithium-ion batteries at industrial scale
Source: Natl Sci Rev. 2026 Jan 10;13(4):nwag017. doi: 10.1093/nsr/nwag017 (PMC12900418; doi:10.1093/nsr/nwag017)
Supplement: nwag017_Supplemental_File [file nwag017_supplemental_file.pdf]

Supplementary information

Flexible direct regeneration of heterogeneous cathode materials of spent lithium-ion batteries at industrial scale

Junxiong Wang [#, a, b], Guanjun Ji [#, b], Haocheng Ji [#, a], Song Liu [b], Junfeng Li [a], Yanfei Zhu [a], Nengzhan Zheng [a], Zheng Liang [*,b], Guangmin Zhou [*,a], Hui-Ming Cheng [*,c,d]

[a]Tsinghua Shenzhen International Graduate School, Tsinghua University, Shenzhen 518055, China;

[b]Frontiers Science Center for Transformative Molecules, School of Chemistry and Chemical Engineering, Shanghai Jiao Tong University, Shanghai 200240, China;

[c]Institute of Technology for Carbon Neutrality, Shenzhen Institute of Advanced Technology, Chinese Academy of Science, Shenzhen 518055, China;

[d]Shenyang National Laboratory for Materials Science, Institute of Metal Research, Chinese Academy of Sciences, Shenyang 110016, China

***Corresponding authors**. E-mails: liangzheng06@sjtu.edu.cn; guangminzhou@sz.tsinghua.edu.cn; hm.cheng@siat.ac.cn

[#] Equally contributed to this work.

(3 texts, 25 figures, 13 tables)

1. Methods in the supplementary file

Raw materials

In order to obtain the degraded cathode material with defined SOH, we assembled 2Ah pouch cells using commercial NCM523 material as cathode materials, and then subjected it to long term cycling to age it to a specific state (50-90% SOH). The cycling performance of the pouch cell before disassembly is shown in **Fig**. S21 in the online Supplementary file. After the residual capacity of singe cell reached the set state, the pouch cell was fully discharged to 2V and then manually disassembled in the laboratory. The outer case was firstly removed, and the electrode sheet was cleaned by dimethyl carbonate (DMC) to remove the residual electrolyte, dried and scraped directly to obtain degraded cathode materials. The obtained cathode material was placed in an NMP solution and stirred to remove as much of the polyvinylidene difluoride (PVDF) binder as possible. The degraded cathode materials with different SOH and different electrochemical performances (**Figs** S22 and S23 in the online Supplementary file) are mainly used to study the mechanism of the repair process as well as the theoretical feasibility of direct repair of mixed cathodes. The other type of degraded NCM523 cathode material used was obtained from Sinochem Hebei Co. by dismantling a large number of actual spent LIBs, the degraded cathode material is also known as black mass. The black mass was obtained from actual spent LIBs after crushing and sorting, with complicated sources and huge quantities. We purchased several tons of black mass for pilot experiment verification, and some basic properties of the black mass are shown in the **Figs** S24 and S25 in the online Supplementary file. The other main chemical

reagents required, such as LiOH, Li_2CO_3, NMP, PVDF, NaOH were purchased from Aladdin reagent Co. and directly used without any treatment.

Battery assembly and electrochemical test

In laboratory experiments, the material performance was tested mainly by coin cells. The cathode material was mixed with the binder and acetylene black (AB) in a ratio of 8:1:1. NMP was added dropwise to form a slurry, which was subsequently coated on a carbon coated aluminum foil. The electrode sheet was then sent into a vacuum oven at 120°C and dried overnight. The dried electrode sheet was cut into 12mm diameter round pieces and used to assemble CR2032 coin cells with a areal loading of about 5 mg cm^{-2}. Lithium chips were used as the anode, the separator was polypropylene (Celgard 2500), and the electrolyte contains $LiPF_6$ in a mixture of equal ratios of ethylene carbonate (EC), diethyl carbonate (DEC), and dimethyl carbonate (DMC). In a larger scale validation of the regenerated cathode materials, pouch cells were used. The specific ratio of slurry was different from coin cells, and the areal loading was approximately 15 mg cm^{-2} on both sides. The anode material used was commercial graphite, and specific parameters such as N/P ratio and cell size are shown in the Supplementary **Table** 8. In order to demonstrate the uniform performance of the regenerated cathode materials, data from at least two samples are provided in the text for each set of cells.

The electrochemical performance of the testing cells, including the rate capabilities and cycling performance, etc., is mainly tested by the Neware testing

system (CT-4008T-5V20mA-164). For coin cells, the voltage range is 2.5-4.3V, the test range for rate capabilities is 0.1-4C, and 0.5C for the long-term cycling test. For pouch cells, the voltage range is 2.8-4.25V, and the cells are activated by two cycles at 0.1C, and then subjected to a long-term cycling test at 1C or 0.5C, and all the tests are carried out at room temperature. Other electrochemical tests of the material, such as cyclic voltammetry (CV) and electrochemical impedance spectroscopy (EIS) tests, were performed using an electrochemical workstation (Biologic, VMP300) based on coin cells. The potential range for the CV test was 3-4.3 V, with a scanning rate gradually increasing from 0.1 to 1 mV s^{-1}, and the EIS test was performed in the frequency range of 0.01-100,000 Hz, with an amplitude of 5 mV.

Characterizations

Infrared spectra (IR) and 2D nuclear magnetic resonance (NMR) spectra of the LiOH-NMP solution were used to analyze the binding form of LiOH with NMP, the IR was tested in the range of 400-4000 cm^{-1}, and the ^{13}C and ^{1}H NMR were tested in the default ranges. In order to study the regeneration mechanism, the whole regeneration process was classified into five processes, i.e., pristine state, after contact lithiation, sintering at 550°C, washing, and sintering at 900°C, and for each of these states the phase was analyzed by XRD (Bruker, D8 advance), morphology was analyzed by SEM (Tescan, Mira3), and changes in surface element state were analyzed by XPS (Thermo Scienctific Escalab QXi) with IR (Thermo Scientific Nicolet is 50) spectroscopy. In order to analyze the lithium-rich phase in the intermediate state in detail, we used a high-resolution transmission electron microscope (HRTEM, FEI Tecnai G2 F30)) and,

with the help of the iDPC technique, we were able to confirm the presence of the lithium-rich regions by directly observing the lithium arrangement. Time of flight secondary ion mass spectrometry (TOF-SIMS) was also used to analyze the changes in lithium content. In-depth XPS was used to analyze the change in nickel valence state with depth, thus demonstrating the change in lithium content laterally. *In situ* XRD of the regeneration process was used to analyze the phase transition of the degraded material as well as the lithium salt transformation process. The Li-Ni anti-sites defects in the degraded cathodes materials with different SOH were obtained from XRD refinement, and their compositional variations were obtained from inductively coupled plasma optical emission spectrometer (ICP-OES) tests. Changes in the weight of the material during the heating process were obtained by thermogravimetric analyzer (TGA).

Technoeconomic analysis

The data for the economic analysis is mainly based on the data obtained during the pilot scale experiment and from a specialized recycling database (EverBatt 2023). We have focused the economic analysis mainly on the comparison with the hydrometallurgical recycling method, which is widely used nowadays, since the raw materials treated are basically the same in both cases. The prices of raw materials and various reagents are based on actual prices, and since the prices of raw materials, especially lithium salts, fluctuate over time, the date when the prices were obtained is also indicated in the Supplementary **Tables** 9-10. By calculating in detail the amount of raw materials used in the different recycling processes and their prices, the material costs of the two

recycling processes can be analyzed. Other costs such as energy consumption, environmental protection and some other fixed costs have also been estimated (Supplementary **Tables** 11-13). By calculating the value of the resulting regenerated products and subtracting the total costs, the profits of the two methods can be accurately compared. The detailed specific calculations are presented in the Supplementary Information. Since this paper has involved large-scale pilot experiments and the prices of various types of raw materials are very close to reality, the results have strong credibility.

2. Details of the direct recycling method for mixed cathode materials

In many previous studies, the parameters of the repair process, especially the amount of supplemental lithium, often need to be finely controlled according to the composition of the degraded material, which is feasible in the laboratory but difficult when confronted with an actual mixture of the degraded material, as the measured compositions are averages and do not represent the true state of the degraded material from different sources. Based on our concept of contact lithiation and synthetic lithiation, we need to ensure that every material particle, whether single crystal or polycrystalline, with a high or low degree of failure, should form a lithium-rich region on its surface, and then complete the self-saturated lithiation repair process through the synthetic lithiation process, thus realizing the on-demand repair of cathode materials with different SOH.

We configured a fixed concentration of LiOH-NMP solution (solid-liquid ratio

1:50), and then mixed each of the three cathode materials with a known SOH with LiOH-NMP solution at the same solid-liquid ratio, respectively, without adjusting the parameters according to the compositions of the different degraded materials. A rough estimation of the molar ratio of lithium in the solution to the degraded cathode material was around 0.8:1, which is far more than the amount of lithium required for the degraded material. The capacity of all three groups of cathode materials was restored to identical levels (with a specific capacity of 155 mAh g^{-1}) after solid-liquid separation, drying and subsequent sintering. We then mixed the three groups of materials in equal proportions and restored them with the same reaction conditions again, and the properties of the resulting products were also relatively homogeneous. This result tentatively proves that our method does not require precise adjustment of the lithium amount according to the composition when dealing with mixed cathode materials, but only ensures that the lithium amount is excessive, which is crucial for the treatment of actual mixed cathode materials.

The complexity of the degraded cathode materials purchased in actual large quantities can be seen from the morphology (Supplementary **Fig**. 25). Due to the differences in the sources, the materials, in addition to the compositional differences, have significantly different morphologies, with some being monocrystalline and some being polycrystalline particles. After our systematic scale-up experiments, under the premise of ensuring the repair effect and cost consideration, we adjusted the concentration of LiOH-NMP solution to ensure that when reacting with the black mass, the molar ratio of lithium in the solution to the degraded material is about 0.15~0.2:1.

The resulting regenerated material has excellent performance, and the material cost of the whole process has been effectively controlled.

The above results based on cathode materials with known SOH and black mass directly procured from unknown sources all indicate that the degraded materials, despite the differences in their initial states, are restored to the same state and have identical performance after repair. This means that the amount of lithium required for the repair of the degraded material should be determined by its own composition, and the process should include a chemical synthetic reaction process with self-saturation properties. As long as the method is well-designed to ensure that, at the microscopic level, all particle surfaces are able to have full contact with lithium, the challenge of repairing mixed material can be solved.

3. Detials of technoeconomic analysis

Materials cost

Based on our previous experience, material cost is the most important part of the recycling process. At present, the most successful commercial recycling method is the hydrometallurgy route, and inorganic acid is usually used. In order to accurately calculate the various reagents needed, we selected the most mature sulfuric acid system at present, and the specific reaction equations are as follows:

Acid leaching: $LiNi_xCo_yMn_zO_2$+3 H^++0.5 H_2O_2 = Li^++2 H_2O+x Ni^{2+}+y Co^{2+}+z Mn^{2+}+ 0.5 O_2 (x+y+z=1)

Chemical precipitation: $2Li^+ + CO_3^{2-} = Li_2CO_3 \downarrow$; x Ni^{2+}+y Co^{2+}+z Mn^{2+}+2 OH^-=

$Ni_xCo_yMn_z(OH)_2\downarrow$

It should be noted that the actual hydrometallurgical recycling process is more complicated than we describe, because the various impurities contained in the real black mass, such as Al, Cu, Fe and graphite, need to be removed by specific processes. However, these steps have no significant impact on the cost, and it is difficult to directly calculate the cost, so they are not included in the cost calculation, which is also the reason why the final calculated profit is slightly higher than the actual income we know.

For the direct recycling method, our estimation of the amount of various reagents used is based on the actual 50 kg scale experiment. The main reagents used include LiOH and NMP, and the solid-liquid ratio in the reaction is approximately 1: 1. Based on the above reactions, we summarized the main types and amounts of chemical reagents used in each recovery method (Supplementary **Table**. 9). Correspondingly, we also calculated the type and mass of the products obtained by each recycling method (Supplementary **Table**. 10). The prices of reagents are determined according to the prices of industrial products, and the data sources are marked in the corresponding tables. Based on the amount and price of reagents, the material cost of each recycling method can be accurately calculated. According to the calculation results, the material cost accounts for 70-80 % of the total cost, which is the most important part of the cost.

Energy consumption

Energy consumption is another important part of the cost of the recycling process. To accurately estimate energy consumption, we must referred to practical production experience. We have consulted some industrial reports, and finally obtained reliable

energy consumption data for hydrometallurgical processes. On the other hand, the energy consumption of the re-synthesis process is directly related to the sintering time. The power consumption for synthesis of a ton of cathode materials in industry is generally 6000-8000 kWh. We estimate the energy consumption of heat treatment proces of the direct recycing based on the difference in heat treatment time, and the results is shown in Supplementary **Table**. 11.

Waste treatment

It is necessary to consider the treatment of waste in practical operation, especially the hydrometallurgical recycling process. In the leaching process, strong organic acid is used, and the solid-liquid ratio of the reaction will be quite high in order to facilitate the reaction, resulting in a large amount of acid-containing wastewater. In the subsequent process, it is often necessary to use alkali to adjust the pH of the solution to achieve the precipitation of Li^{+}. These processes will produce a large amount of acid and alkali wastewater, which needs to be treated carefully, resulting in a certain cost. Other possible wastes also include waste gas and dust. After referring to some industry report data, we initially estimated the waste treatment costs of hydrometallurgical methods. The direct recycling method does not produce any acid and alkali wastewater or other waste liquid due to the small solid-liquid ratio, so the waste treatment costs can be ignored (Supplementary **Table**. 12).

Production fee

In the practical recycling operation, there will be some other costs, including equipment depreciation, labor, consumables, and other parts, these costs are difficult to

directly estimate. Considering that the requirements for the equipment of different recycling methods are almost identical, so the production costs of them are calculated the same here. We mainly refer to the relevant data in the EverBatt2023 model to estimate production costs, and the specific result is shown in Supplementary **Table**. 13.

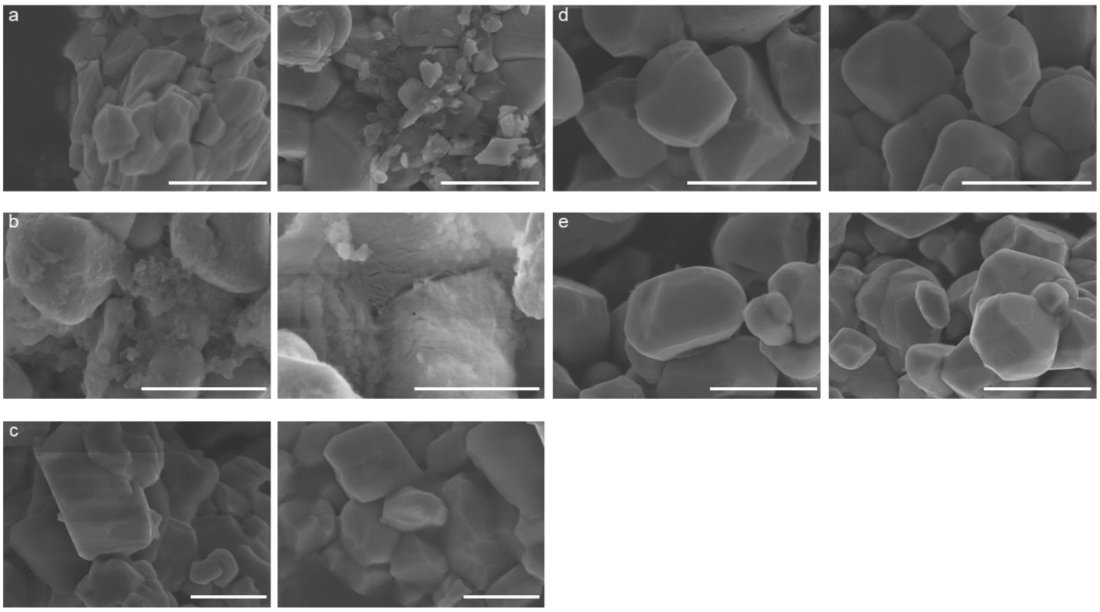

Fig. 1 SEM images of NCM523 samples at different stages during regeneration, **a**, pristine degraded NCM, **b**, after contact lithiation, **c**, sintering at 550°C, **d**, washing, **e**, sintering at 900°C (scale bar=1 μm). The morphology of the original degraded cathode material was irregular, with some residual lithium salts and cracks on the surface of the particles. After the reaction with LiOH-NMP solution, the surface of the particles appeared uniformly distributed coatings, and after heating at 550°C, most of the surface coatings disappeared, and the surface of the particles after washing was more polished, and then after heating at 900°C, the change of the morphology of the particles was not obvious.

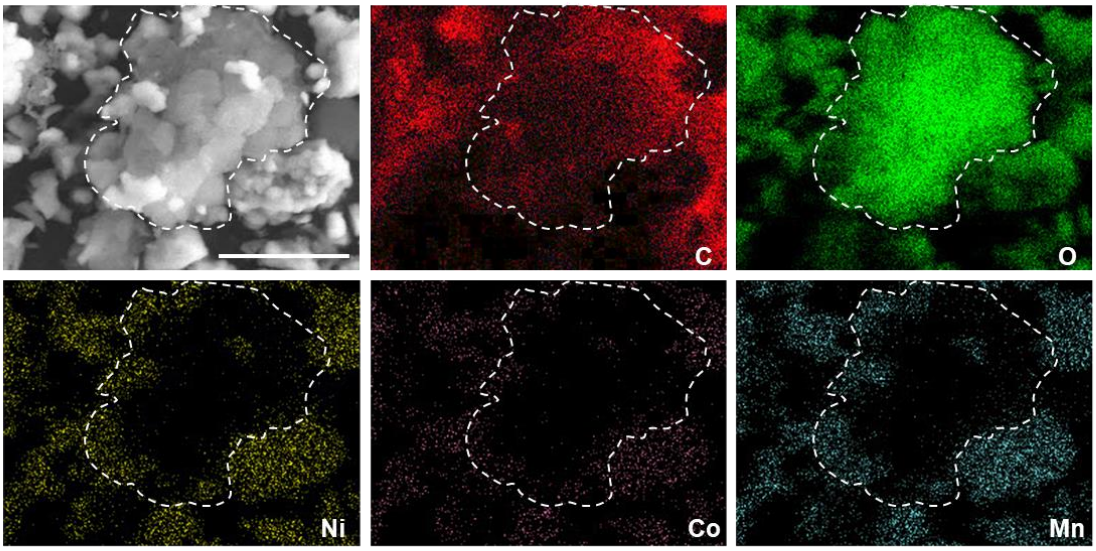

Fig. 2 Element distribution of NCM523 samples after contact lithiation (scale bar=5 μm). The C signal on the surface of the particles comes from the C in the LiOH-NMP molecules, and the distribution of the C element is almost all over the surface of the particles, indicating that the LiOH-NMP molecules are in good contact with all of the NCM cathode particles.

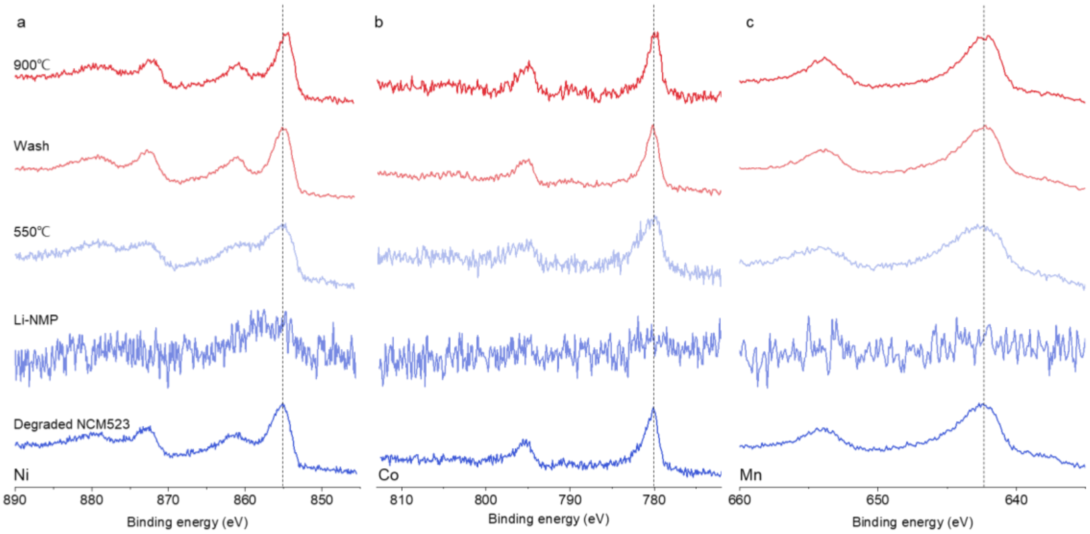

Fig. 3 XPS spectra of NCM523 samples at different stages during regeneration, **a**, Ni 2p, **b**, Co 2p, **c**, Mn 2p. From the XPS results, the main peak of Ni is shifted as the regeneration process proceeds because the re-intercalation of lithium leads to a change

in the valence state of Ni. The peak position of Mn is basically unchanged because it does not participate in the redox reaction. The peak position of Co in the intermediate state has a small change, but its peak position is basically the same as the original position after the final regeneration.

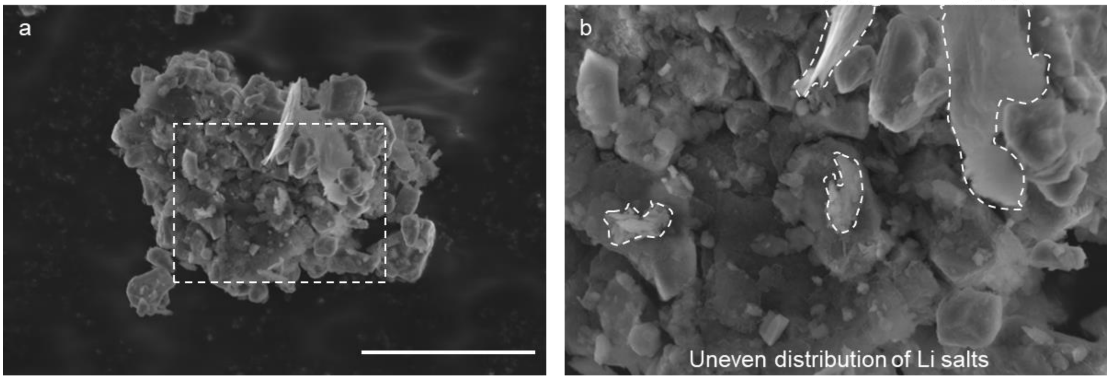

Fig. 4 SEM images of degraded cathode samples after direct mechanical mixing with solid LiOH (scale bar=5 μm). It can be clearly seen that the flakes of LiOH have agglomeration, and the contact with the NCM cathode particles is very uneven, which means that many particles will not be able to contact with the lithium source in the subsequent lithiation process, resulting in a poor overall repair effect.

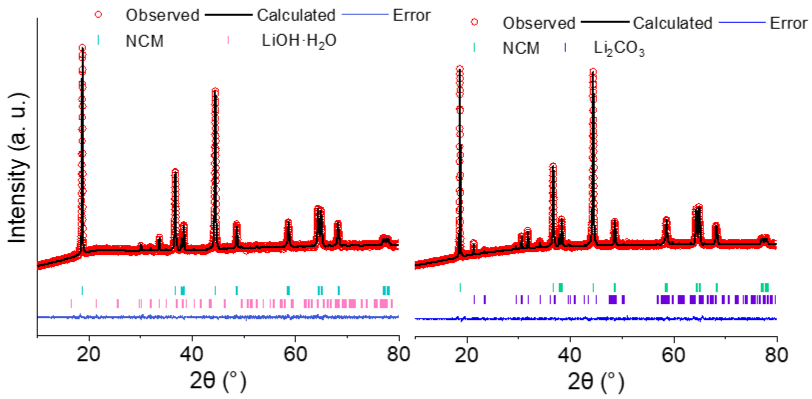

Fig. 5 XRD refinement results of NCM523 samples after contact lithiation and 550°C

sintering. The results of XRD refinement show that the intact LiOH structure is still retained in the LiOH-NMP molecule, while a part of excessive LiOH is gradually converted to Li_2CO_3 upon heating in air.

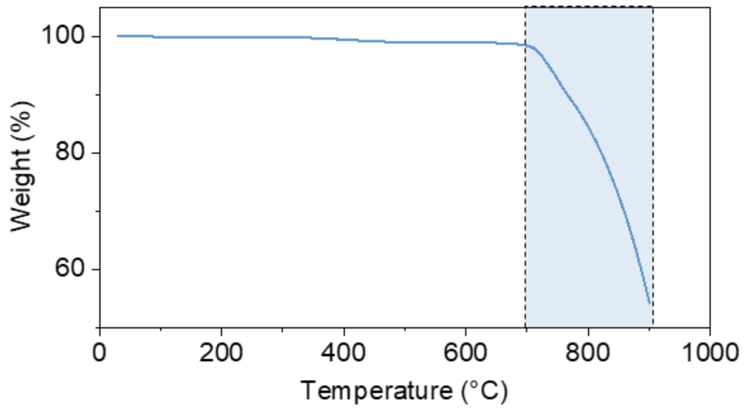

Fig. 6 Thermogravimetric curves of pure Li_2CO_3. Li_2CO_3 must be heated above 700℃ to gradually thermally decompose and produce Li_2O, according to the TG result.

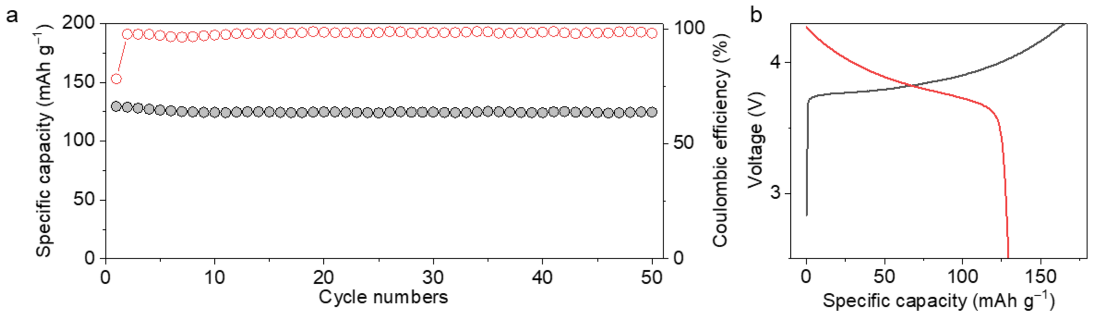

Fig. 7 Electrochemical performance of NCM523 samples after contact lithiation and 550℃ sintering, **a**, cycling performance, **b**, charging/discharging curves. After contact lithiation and sintering at 550℃, the material has been repaired in terms of morphology and components, but its electrochemical performance is not fully recovered, indicating that the microstructure of the material has not been totally repaired.

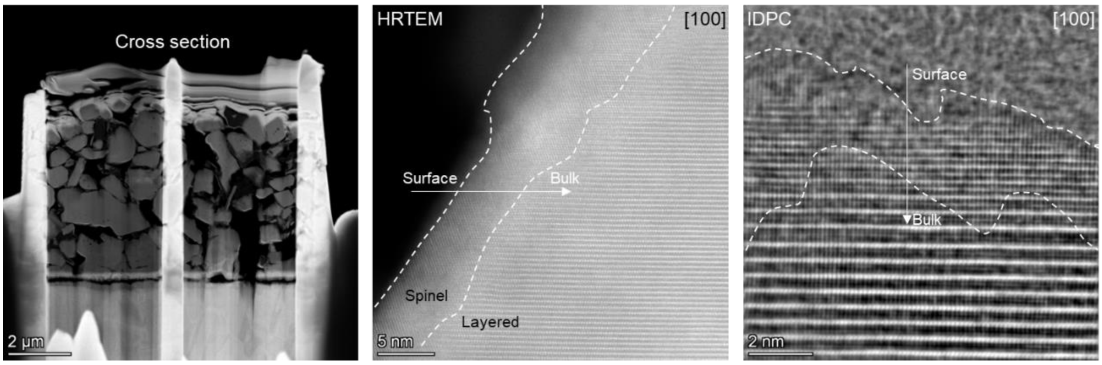

Fig. 8 Analysis of surface-to-bulk phase transitions in degraded cathode materials. After cutting the particles apart using a focused ion beam, along the cross-section of the degraded cathode material, it is clearly observed that there is a difference in the atomic arrangement between the surface and the bulk, which is caused by the phase transition originated from the change of surface composition over a long period of cycling.

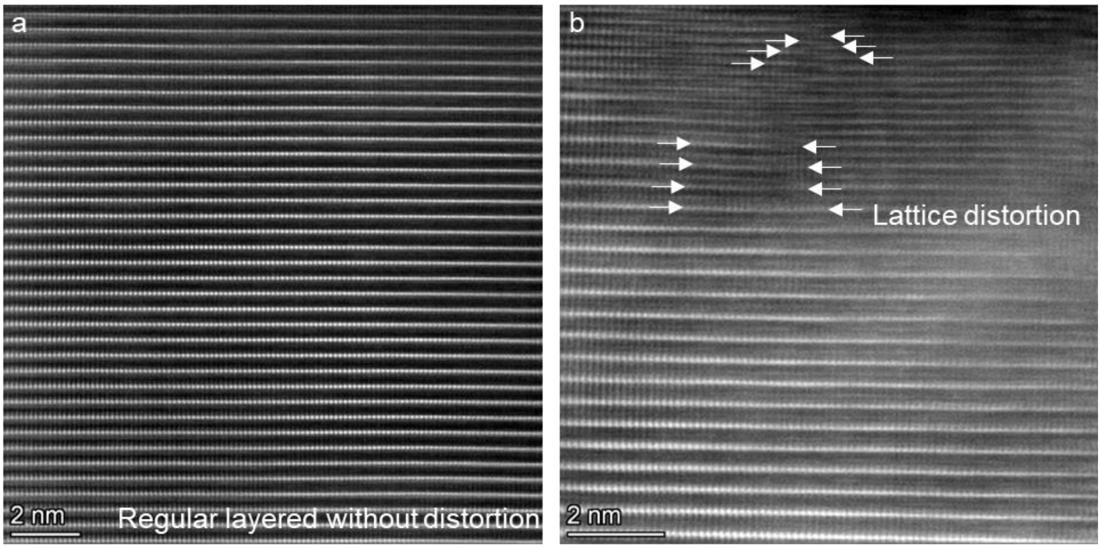

Fig. 9 Comparative analysis of the atomic arrangement of the regular layered structure and the lattice distortion caused by stacking fault of Li in the interlayer.

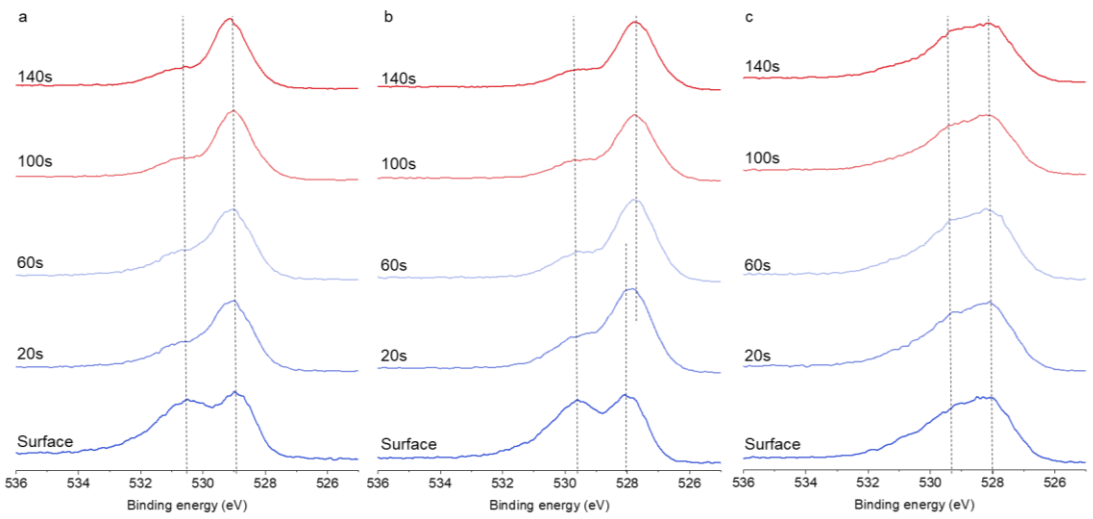

Fig. 10 In-depth XPS spectra of O 1s of different NCM cathode samples, **a**, pristine degraded NCM, **b**, after contact lithiation and 550°C sintering, **c**, sintering at 900°C. The O 1s profile of the surface of the degraded NCM cathode is different from that of the bulk, but the internal state is basically the same; After contact lithiation and heating at 550°C, the O 1s profiles of the surface and near-surface change, while further etching into the bulk, the state is basically unchanged again, which is consistent with the results of Ni 2p, suggesting that at this point, the near-surface of the material changes with the intercalation of lithium, but more internally, there is no change; while the completely regenerated material has no change in the O 1s profile from inside to outside.

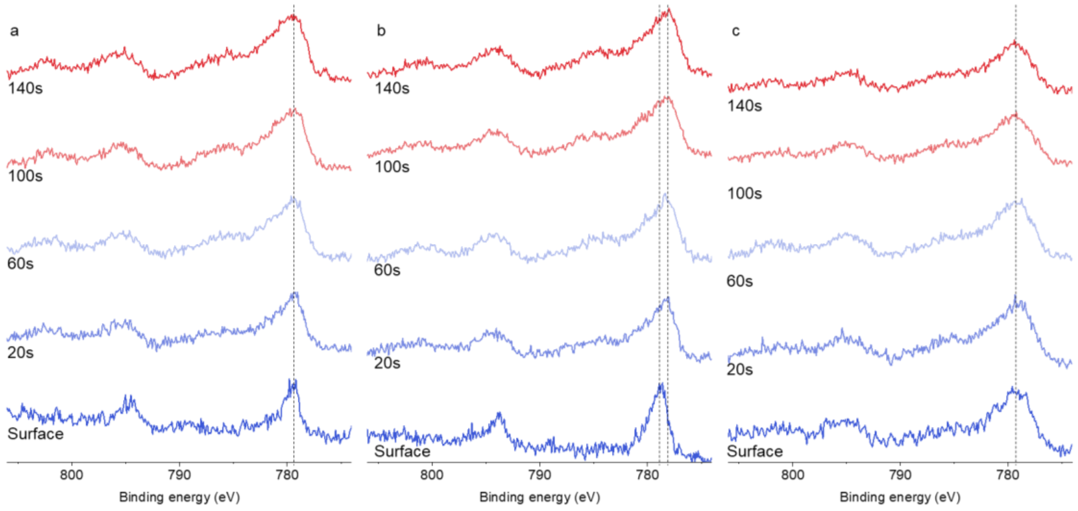

Fig. 11 In-depth XPS spectra of Co 2p of different NCM cathode samples, **a**, pristine degraded NCM, **b**, after contact lithiation and 550°C sintering, **c**, sintering at 900°C. The Co 2p profiles of the pristine degraded NCM and the final regenerated samples are basically the same, but for the intermediate state samples, there is a certain shift from the outside to the inside, which may be related to the intercalation of lithium.

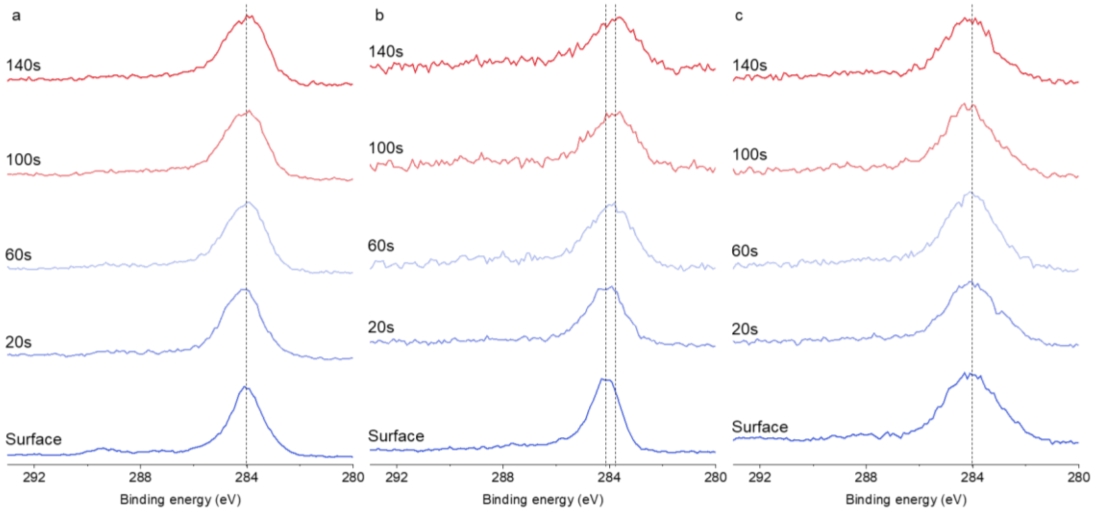

Fig. 12 In-depth XPS spectra of C 1s of different NCM cathode samples, **a**, pristine degraded NCM, **b**, after contact lithiation and 550°C sintering, **c**, sintering at 900°C. The C 1s profiles of the pristine degraded NCM and the final regenerated samples are

basically the same, but for the intermediate state samples, there is a certain shift from the outside to the inside, which indicates that the inner and outer states of the samples are not homogeneous at this time.

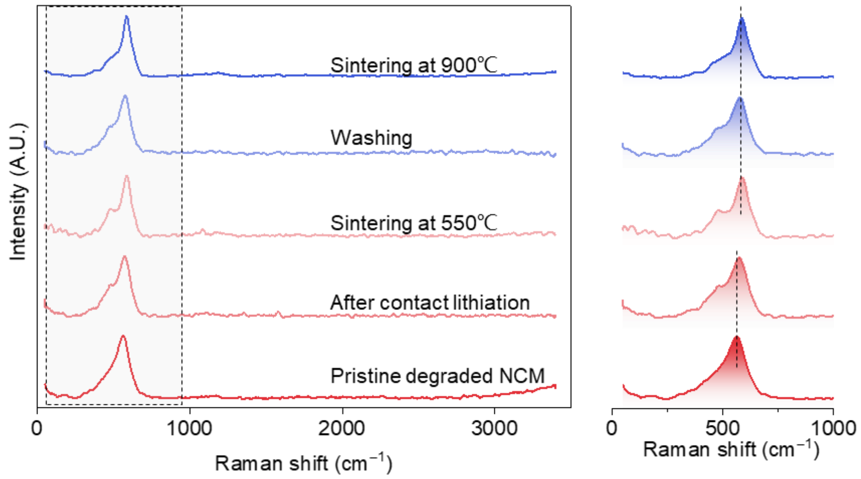

Fig. 13 Raman spectra of NCM523 samples at different stages during regeneration. The main peak shifted after contact lithiation and 550℃ sintering, which was caused by lithiation of the cathode material.

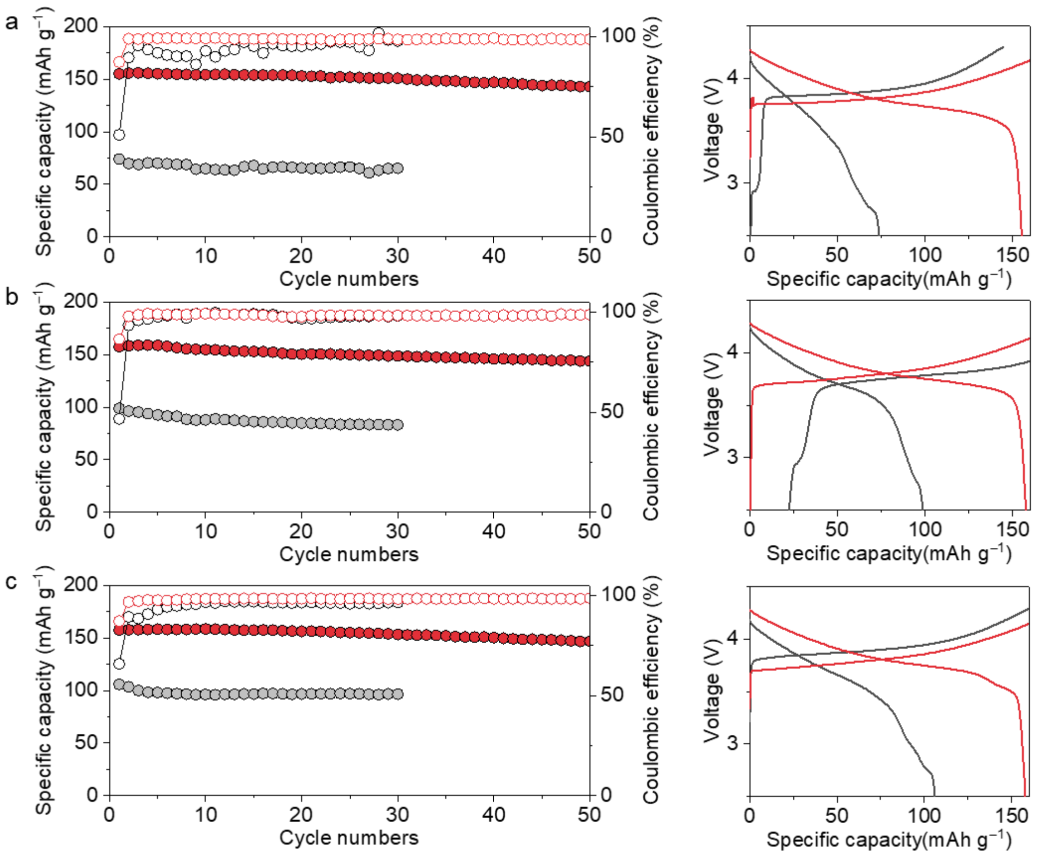

Fig. 14 Comparison of the original and repaired electrochemical performance of degraded cathode materials with different SOH, **a**, 50%, **b**, 70%, **c**, 90%.

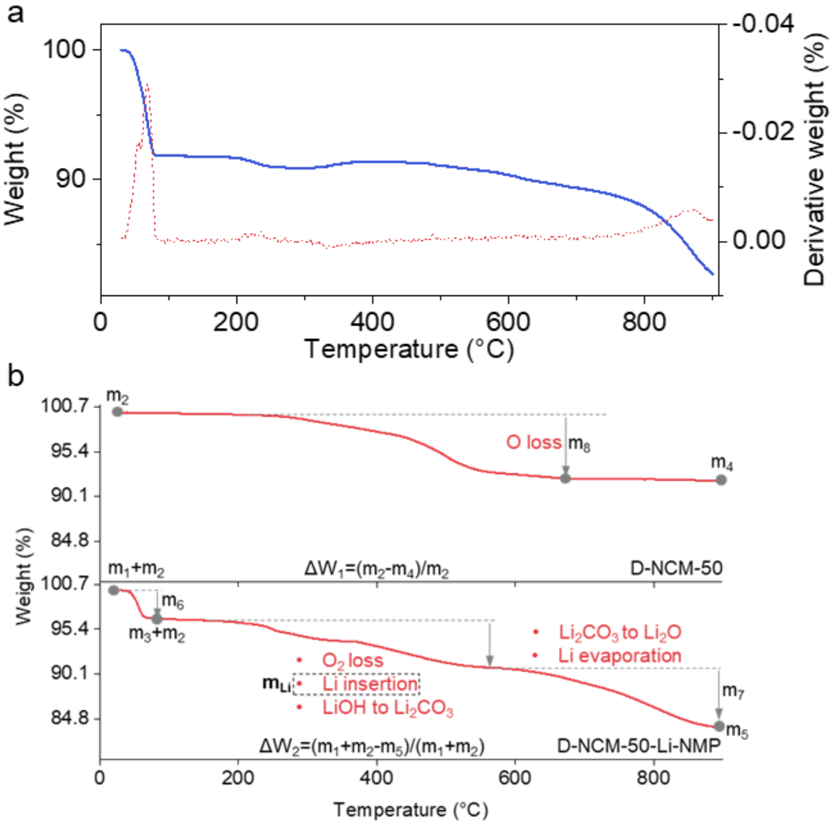

Fig. 15 **a**, Thermogravimetric curves of commercial NCM materials after reaction with LiOH-NMP solution. **b**, The correspondence between mass changes during the lithiation process and TG analysis results in Figs. 3 and f. Since the lithium component is not missing in the commercial NCM cathode material, it cannot undergo a synthetic reaction with LiOH-NMP molecules, and all lithium salts are completely volatilized with a weight loss of 17.3%, which is consistent with the trend that the lower the degree of failure, the greater the weight loss after heating.

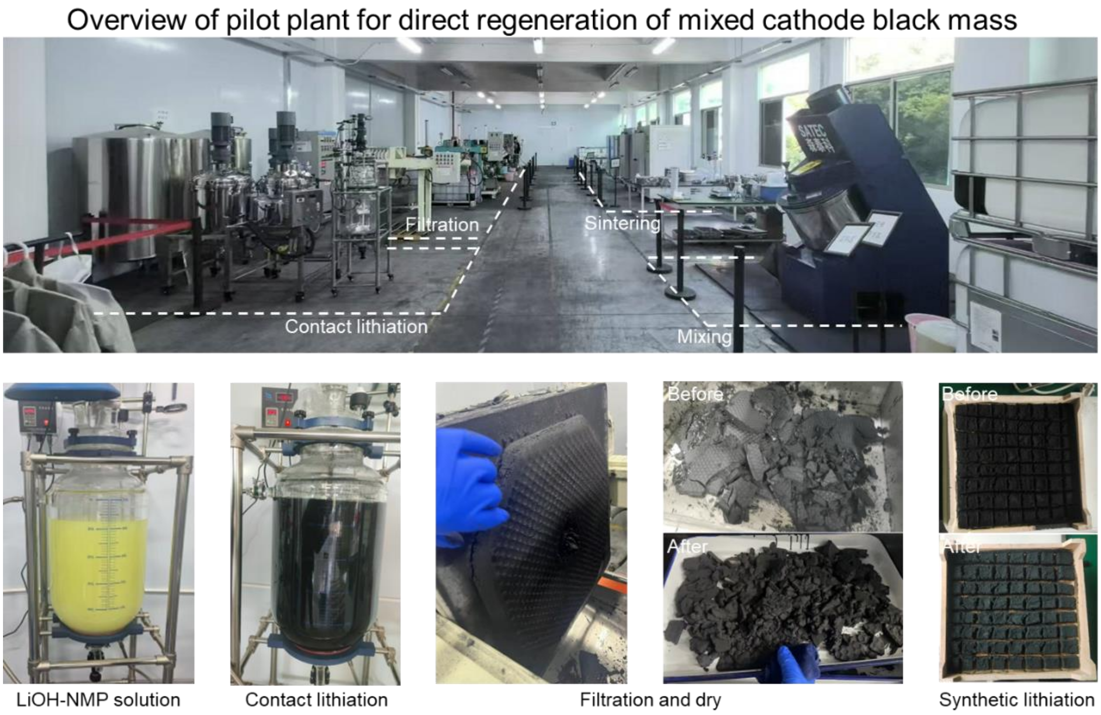

Fig. 16 Photos of the pilot plant for direct regeneration of mixed cathode black mass.

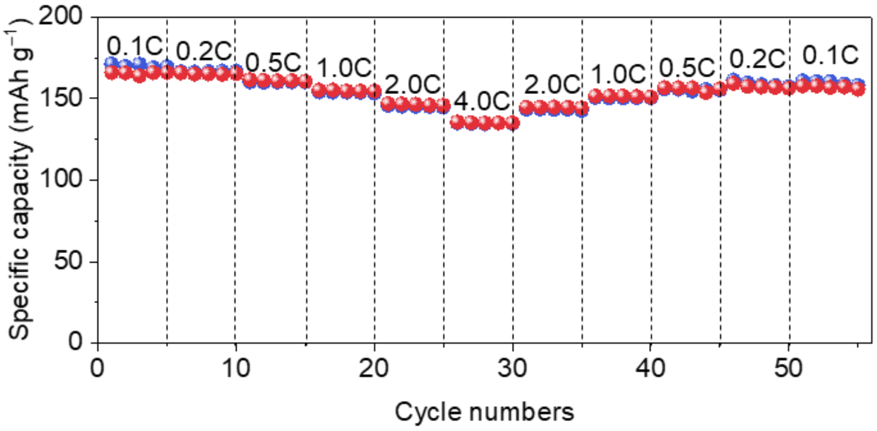

Fig. 17 Rate capabilities of regenerated NCM cathode materials.

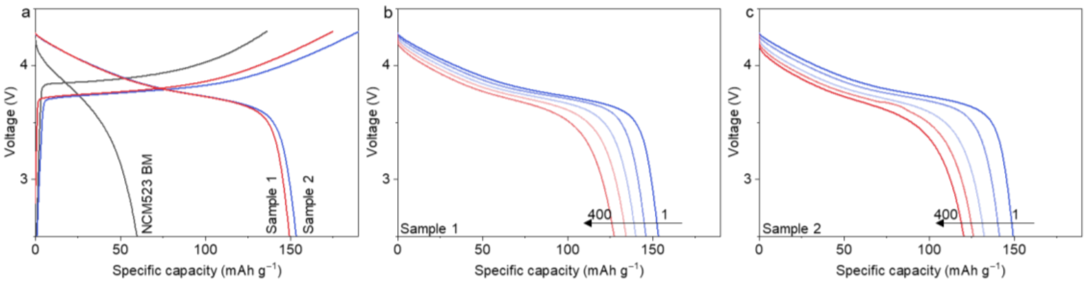

Fig. 18 Charge/discharge curves of NCM523 black mass and regenerated samples at **a**, 1st cycle, **b-c**, different cycles.

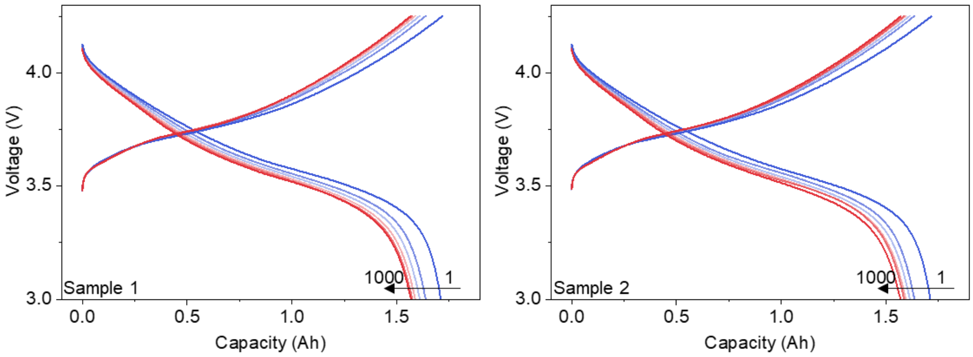

Fig. 19 Charge/discharge curves of pouch cell samples using regenerated NCM as cathode materials at different cycles.

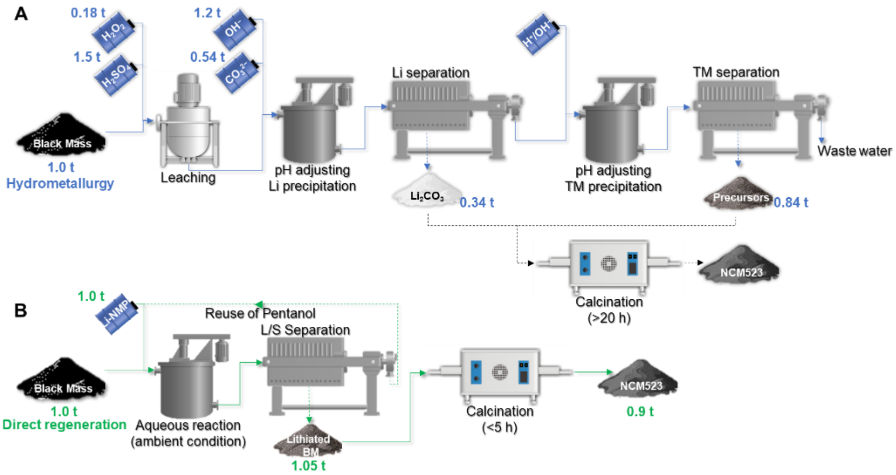

Fig. 20 Schematic comparing the process of the hydrometallurgical recycling method with the direct recycling method in this paper.

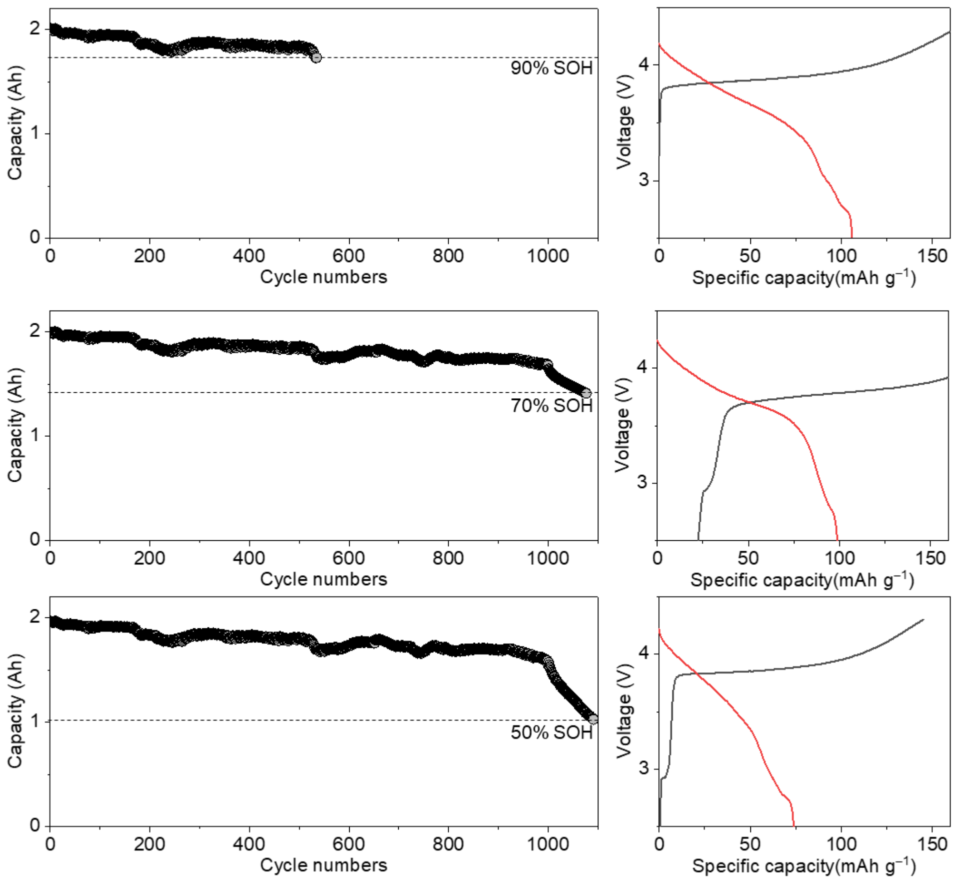

Fig. 21 Raw cycling data and charge/discharge curves of pouch cells with different SOH.

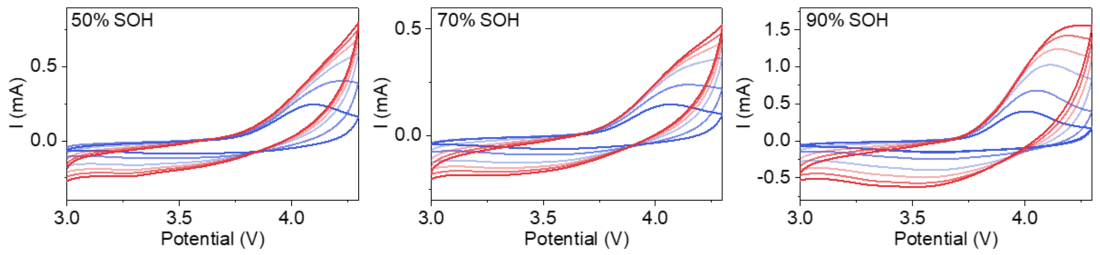

Fig. 22 CV curves of degraded cathode materials with different SOH.

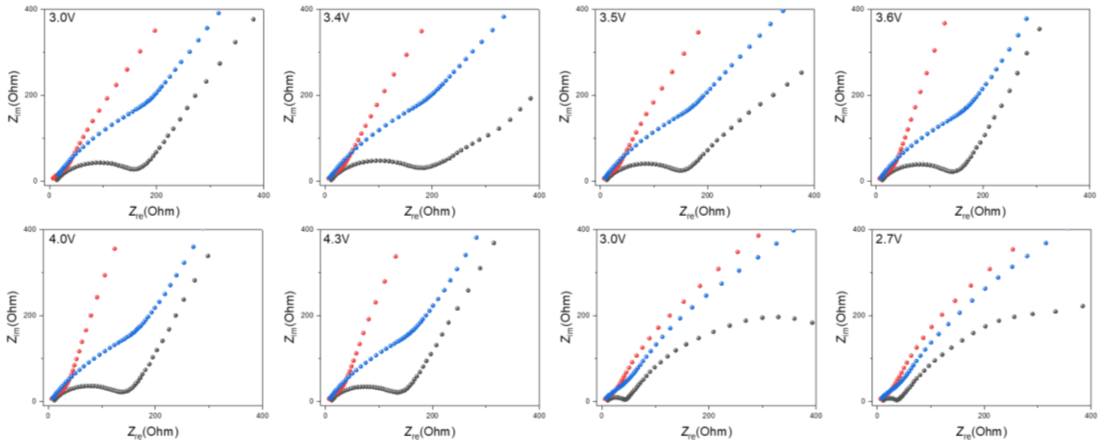

Fig. 23 EIS spectra of degraded cathode materials with different SOH at different potentials.

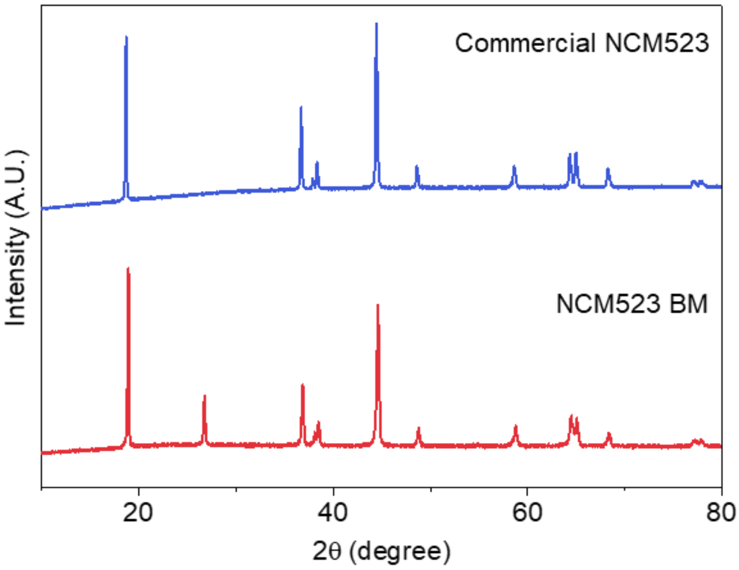

Fig. 24 XRD patterns of NCM523 black mass.

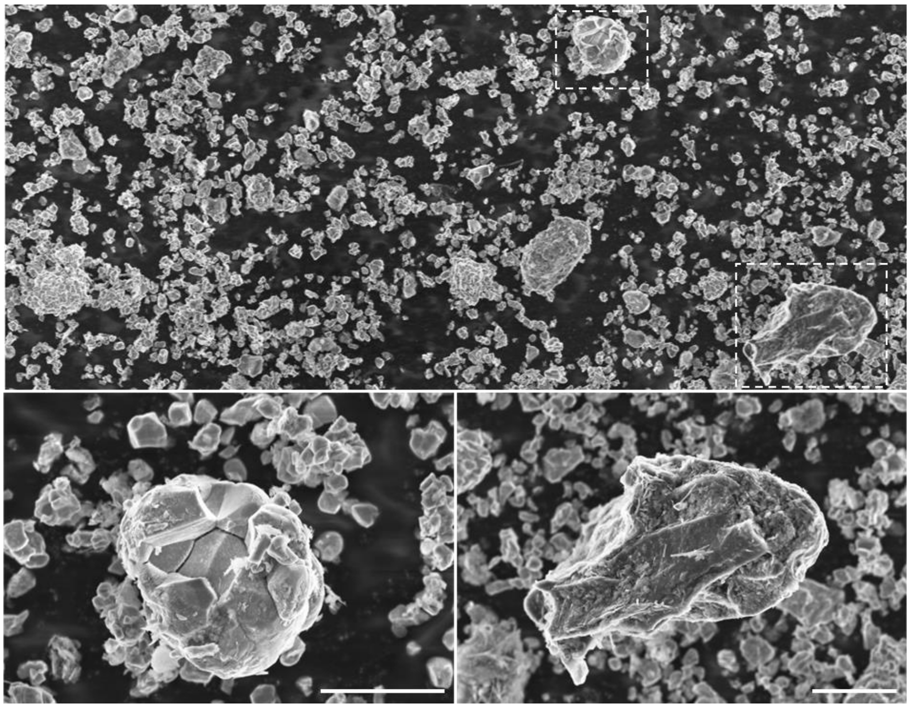

Fig. 25 SEM images of NCM523 black mass (Scale bar=5 μm).

Table 1 XRD Refinement Results of degraded cathode after contact lithiation

**Mixed-SNCM-L-N** (Space Group R-3m)						
	**Atom**	**x**	**y**	**z**	**Occ.**	**Biso.**
**Atomic Occupancies**	Li_{3a}	0.0000	0.0000	0.0000	0.95(2)	1.5373(9)
	Ni_{3a}	0.0000	0.0000	0.0000	0.04(9)	
	Ni_{3b}	0.0000	0.0000	0.5000	0.45(2)	1.7467(5)
	Li_{3b}	0.0000	0.0000	0.5000	0.04(9)	
	Mn_{3b}	0.0000	0.0000	0.5000	0.3	
	Co_{3b}	0.0000	0.0000	0.5000	0.2	
	O_{6c}	0.0000	0.0000	0.7568(1)	1.0	1.0151(4)
**Lattice Parameters**	***a* / Å**		***c* / Å**		**V / Å^3**	
	2.8659(2)		14.2808(1)		101.57(9)	
**Li/Ni Antisite**	4.9%					
**Agreement Factors**						
χ^2	1.30%		R_p	1.45%	R_{wp}	1.79%

Note: The secondary phase is $LiOH\cdot H_2O$ (Space Group C12/m1), with a phase ratio of 16.55%.

Table 2 XRD Refinement Results of degraded cathode after contact lithiation and 550°C sintering

**Mixed-SNCM-L-N-550** (Space Group R-3m)						
	**Atom**	**x**	**y**	**z**	**Occ.**	**Biso.**
**Atomic Occupancies**	Li_{3a}	0.0000	0.0000	0.0000	0.93(4)	1.8653(2)
	Ni_{3a}	0.0000	0.0000	0.0000	0.06(6)	
	Ni_{3b}	0.0000	0.0000	0.5000	0.43(4)	1.2849(4)
	Li_{3b}	0.0000	0.0000	0.5000	0.06(6)	
	Mn_{3b}	0.0000	0.0000	0.5000	0.3	
	Co_{3b}	0.0000	0.0000	0.5000	0.2	
	O_{6c}	0.0000	0.0000	0.7545(1)	1.0	1.1522(1)
**Lattice Parameters**	***a* / Å**		***c* / Å**		**V / Å³**	
	2.8709(5)		14.2414(3)		101.65(3)	
**Li/Ni Antisite**	6.6%					
**Agreement Factors**						
χ^2	1.39%		R_p 1.77%		R_{wp} 1.94%	

Note: The secondary phase is Li_2CO_3 (Space Group C12/c1), with a phase ratio of 12.03%.

Table 3 XRD Refinement Results of degraded cathode with 90% SOH

**SNCM-90** (Space Group R-3m)						
**Atomic Occupancies**	**Atom**	**x**	**y**	**z**	**Occ.**	**Biso.**
	Li_{3a}	0.0000	0.0000	0.0000	0.80(6)	1.2775(6)
	Ni_{3a}	0.0000	0.0000	0.0000	0.05(4)	
	Ni_{3b}	0.0000	0.0000	0.5000	0.44(6)	1.7664(3)
	Li_{3b}	0.0000	0.0000	0.5000	0.05(4)	
	Mn_{3b}	0.0000	0.0000	0.5000	0.3	
	Co_{3b}	0.0000	0.0000	0.5000	0.2	
	O_{6c}	0.0000	0.0000	0.7563(6)	1.0	1.3452(1)
**Lattice Parameters**	***a* / Å**		***c* / Å**		**V / Å^3**	
	2.8602(3)		14.3109(2)		101.38(8)	
**Li/Ni Antisite**	5.4%					
**Agreement Factors**						
χ^2	1.43%	**R_p**	1.78%	**R_{wp}**	1.84%	

Table 4 XRD Refinement Results of degraded cathode with 70% SOH

**SNCM-70** (Space Group R-3m)						
	**Atom**	**x**	**y**	**z**	**Occ.**	**Biso.**
**Atomic Occupancies**	Li_{3a}	0.0000	0.0000	0.0000	0.77(2)	1.5375(2)
	Ni_{3a}	0.0000	0.0000	0.0000	0.05(8)	
	Ni_{3b}	0.0000	0.0000	0.5000	0.44(2)	1.5843(2)
	Li_{3b}	0.0000	0.0000	0.5000	0.05(8)	
	Mn_{3b}	0.0000	0.0000	0.5000	0.3	
	Co_{3b}	0.0000	0.0000	0.5000	0.2	
	O_{6c}	0.0000	0.0000	0.7569(7)	1.0	1.2754(8)
**Lattice Parameters**	***a* / Å**		***c* / Å**		**V / Å^3**	
	2.8590(3)		14.3171(5)		101.34(7)	
**Li/Ni Antisite**	5.8%					
**Agreement Factors**						
χ^2	1.38%		**R_p** 1.42%		**R_{wp}** 1.69%	

Table 5 XRD Refinement Results of degraded cathode with 50% SOH

**SNCM-50** (Space Group R-3m)						
	**Atom**	**x**	**y**	**z**	**Occ.**	**Biso.**
**Atomic Occupancies**	Li_{3a}	0.0000	0.0000	0.0000	0.73(2)	1.2756(7)
	Ni_{3a}	0.0000	0.0000	0.0000	0.06(4)	
	Ni_{3b}	0.0000	0.0000	0.5000	0.43(2)	1.6934(4)
	Li_{3b}	0.0000	0.0000	0.5000	0.06(4)	
	Mn_{3b}	0.0000	0.0000	0.5000	0.3	
	Co_{3b}	0.0000	0.0000	0.5000	0.2	
	O_{6c}	0.0000	0.0000	0.7579(5)	1.0	1.3742(5)
**Lattice Parameters**	***a* / Å**		***c* / Å**		**V / Å^3**	
	2.8549(6)		14.3410(3)		101.34(7)	
**Li/Ni Antisite**	6.4%					
**Agreement Factors**						
χ^2	1.35%	**R_p**	1.37%	**R_{wp}**	1.66%	

Table 6 Compositional analysis of different NCM samples

Samples	Concentration（mg/kg）				Li/TM ratio
	Mn	Li	Ni	Co	
SOH-50	151636	53666	285469	114643	0.809
SOH-70	160117	53862	275198	109852	0.824
SOH-90	161672	55876	283810	112130	0.836
Black mass	120564	48596	226292	89231	0.91

Table 7 Comparison of cycling performance of previously reported regenerated NCM cathodes and this work.

Cathode type	Capacity retention rate	Cell type	References
NCM523	94.3%@500 cycles	Pouch cell	32
NCM523	92.7%@1000 cycles	Pouch cell	33
Ni66	94.0%@500 cycles	Pouch cell	34
NCM811	90.96%@500 cycles	Pouch cell	35
NCM523	80.0%@450 cycles	Pouch cell	36
NCM111	81.2%@500 cycles	Coin cell	37
NCM622	86.0%@200 cycles	Coin cell	38
Ni83	82.1%@200 cycles	Coin cell	39
NCM523	91.7%@100 cycles	Coin cell	40
NCM523	93.5%@150 cycles	Coin cell	41
NCM523	94.5%@100 cycles	Coin cell	42
NCM622	87.5%@100 cycles	Coin cell	43
NCM523	89.6%@200 cycles	Coin cell	44
NCM523	90.6%@150 cycles	Coin cell	45
NCM622	83.9%@200 cycles	Coin cell	46
NCA	85.1%@250 cycles	Coin cell	47
NCM523	83.2%@300 cycles	Coin cell	48
NCM622	94.3%@240 cycles	Coin cell	49
This work			
NCM523	78.3%@500 cycles	Coin cell	
NCM523	93.1%@1000 cycles	Pouch cell	

Table 8 Specific parameters of homemade pouch cell.

	Cathode	Anode
Active materials	Regenerated NCM523	Graphite
Cell dimension (cm×cm)	7.2×6.2	7.5×6.4
Compositions	96:1:0.5:2.5 (NCM523: SP: CNT: PVDF)	95:1:0.5:1.5:2 (Graphite: SP: CNT: CMC: SBR)
One-side loading (mg cm^{-2})	15	6.4
Two-side loading (mg cm^{-2})	30	12.8
Stacking	11	12
Negative/positive (N/P) ratio	1.09	
Designed capacity (mAh)	1800	
Amount of electrolyte	6.3 (3.5 g/Ah)	

Table 9 Main materials used in different recycling processes*.

Materials	Prices ($/ t)	Usage for hydro- (t)	Usage for Direct (t)
NCM 523 black mass	7072	1.0	1.0
H_2SO_4	49.7	1.5	/
H_2O_2	248.6	0.18	/
NaOH	490	1.2	1.0
Na_2CO_3	249	0.54	/
NMP	1381	/	1.0
LiOH	8675	/	0.015

* The prices of each kind of materials are obtained from following sources: http://www.nebrpc.cn/c/price.html; https://www.100ppi.com. (Access date: Oct 22, 2024)

Table 10 Main products of different recycling processes*.

Materials	Prices ($/t)	Production from Hydro- (t)	Production from direct (t)
Li_2CO_3	10111	0.34	/
NiMnCo precursors	8771.5	0.84	/
NCM523	14849	0.85	0.9

* The prices of each kind of materials are obtained from following sources: http://www.nebrpc.cn/c/price.html. (Access date: Oct 22, 2024)

Table 11 Energy consumptions of different recycling processes*.

Category	Energy consumption during recycle (kWh)	Energy consumption during resynthesis (kWh)	Energy cost ($/t)
Hydro-	~2000	~7500	957.6
Direct	~1200	~2000	322.6

* The estimation of energy consumption is based on the following industry report: https://pdf.dfcfw.com/pdf/H3_AP202208161577268907_1.pdf?1660666880000.pdf; https://www.vzkoo.com/read/69529e79f082a5cd5920650d8c6dd6e5.html.

Table 12 Environmental protection fees of different recycling processes*.

Category	Environmental protection fee ($/t)
Hydro-	252
Direct	/

* The estimation of energy consumption is based on the following industry report: https://pdf.dfcfw.com/pdf/H3_AP202208161577268907_1.pdf?1660666880000.pdf

Table 13 Production cost of different recycling processes.

Type	Cost ($/t)
Pretreatment	500
Equipment maintenance	15
Depreciation	65
Labor	140
Other direct cost	275
Total	995
